# Interleukin enhancer‐binding factor 2 promotes cell proliferation and DNA damage response in metastatic melanoma

**DOI:** 10.1002/ctm2.608

**Published:** 2021-10-14

**Authors:** Xiaoqing Zhang, Matias A. Bustos, Rebecca Gross, Romela Irene Ramos, Teh‐Ling Takeshima, Gordon B. Mills, Qiang Yu, Dave S. B. Hoon

**Affiliations:** ^1^ Department of Translational Molecular Medicine Providence Saint John's Health Center Saint John's Cancer Institute Santa Monica California; ^2^ Department of Cell Development and Cancer Biology Knight Cancer Institute Oregon Health and Science University Portland Oregon; ^3^ Agency for Science Technology and Research (A*STAR) Genome Institute of Singapore Biopolis Singapore

**Keywords:** ATM, DNA damage response, ILF2, metastatic melanoma, RAD50, U2AF2

## Abstract

**Background:**

1q21.3 amplification, which is frequently observed in metastatic melanoma, is associated with cancer progression. Interleukin enhancer‐binding factor 2 (*ILF2*) is located in the 1q21.3 amplified region, but its functional role or contribution to tumour aggressiveness in cutaneous melanoma is unknown.

**Methods:**

In silico analyses were performed using the TCGA SKCM dataset with clinical annotations and three melanoma microarray cohorts from the GEO datasets. RNA in situ hybridisation and immunohistochemistry were utilised to validate the gene expression in melanoma tissues. Four stable melanoma cell lines were established for in vitro ILF2 functional characterisation.

**Results:**

Our results showed that the *ILF2* copy number variation (CNV) is positively correlated with *ILF2* mRNA expression (*r* = 0.68, *p *< .0001). Additionally, *ILF2* expression is significantly increased with melanoma progression (*p *< .0001), and significantly associated with poor overall survival for metastatic melanoma patients (*p *= .026). The overexpression of ILF2 (ILF2‐OV) promotes proliferation in metastatic melanoma cells, whereas ILF2 knockdown decreases proliferation by blocking the cell cycle. Mechanistically, we demonstrated the interaction between ILF2 and the splicing factor U2AF2, whose knockdown reverses the proliferation effects mediated by ILF2‐OV. Stage IIIB–C melanoma patients with high *ILF2*‐*U2AF2* expression showed significantly shorter overall survival (*p *= .024). Enhanced ILF2/U2AF2 expression promotes a more efficient DNA‐damage repair by increasing *RAD50* and *ATM* mRNA expression. Paradoxically, metastatic melanoma cells with ILF2‐OV were more sensitive to ATM inhibitors.

**Conclusion:**

Our study uncovered that ILF2 amplification of the 1q21.3 chromosome is associated with melanoma progression and triggers a functional downstream pathway in metastatic melanoma promoting drug resistance.

## Graphical abstract

• ILF2‐U2AF2 regulates cell proliferation and DNA damage response by upregulating RAD50 and ATM.

• Concurrent enhanced ILF2‐U2AF2 expression is associated with a worse overall survival.

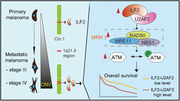

## 1 BACKGROUND

Cutaneous melanoma has an increasing incidence rate in Western countries, especially in the United States.^1^, ^2^ Patients with melanoma metastasis to distant organs, such as visceral organs or brain, exhibit a very poor prognosis with a 5‐year survival rate of approximately 35%.^3^, ^4^ The first‐line systemic therapy options for stage IV metastatic melanoma patients are immune checkpoint inhibitors and/or targeted therapies.^5^, ^6^, ^7^ However, a high percentage of metastatic melanoma patients develop resistance and progress within 5 years of treatment. For patients with advanced stage melanoma, chemotherapeutics such as temozolomide (TMZ) are approved for treatment.^8^ Few patients with advanced disease have successful responses to TMZ treatment. Therefore, a better understanding of the functional and genomic events that drive melanoma progression is important to determine patients that are at higher risk of developing aggressive metastatic disease and to identify potential novel therapeutic targets.^9^, ^10^, ^11^, ^12^, ^13^

Amplification of the 1q21.3 region has been observed in several cancers and is associated with progression.^14^, ^15^, ^16^, ^17^ In a previous study, we showed that 1q21.3 amplification is a biomarker for breast cancer disease progression and response to treatment; where ∼10–30% of breast cancer patients develop 1q21.3 amplification in primary tumours and the percentage increases ∼70% in metastatic tumours.^14^ In multiple myeloma, 1q21 amplification enhances ILF2 expression, which promotes resistance to melphalan.^16^ Previously we identified 1q21.3 amplification in metastatic melanoma cell lines, tumour tissues and circulating tumour cells (∼80% for stage IIIB–C patients).^18^ However, the role of specific genes located in the 1q21.3 amplified region on metastatic melanoma and the downstream affected pathways are not well‐defined.

In the present study, we characterised the function of the *ILF2* gene, which is located in the 1q21.3 region. *ILF2* was initially identified as a transcription factor required for interleukin 2 gene expression,^19^ but later ILF2 was shown to regulate RNA splicing and DNA repair process.^16^, ^20^ Differential expression of ILF2 has previously been observed in some cancers, but the ILF2 function is not clear.^16^, ^21^, ^22^, ^23^ Our studies showed that ILF2 levels were significantly increased during melanoma progression and positively correlated with gene CNV. We demonstrated that ILF2 interacts with the splicing factor U2 small nuclear ribonucleoprotein auxiliary factor 2 (U2AF2). U2AF2 expression significantly increased with the stage of melanoma. Furthermore, we showed that ILF2‐U2AF2 complex enhanced RAD50 and ATM expression in metastatic melanoma cells. Consequently, ILF2‐U2AF2 promoted resistance to TMZ, but also enhanced the sensitivity to ATM inhibitor (ATMi) in metastatic melanoma.

## 2 MATERIALS AND METHODS

### 2.1 Melanoma cell lines

Established metastatic melanoma cell lines from SJCI were attained from melanoma patients who received elective surgery (DP‐0574, IM‐0223 and M‐204).^24^ The cell lines were cultured in RPMI‐1640 and supplemented with 10 mM HEPES, 10% heat‐inactivated foetal bovine serum (FBS) and 1% penicillin‐streptomycin (complete medium). All human cell lines have been authenticated using short tandem repeat (STR) profiling within the last three years. All experiments were performed with mycoplasma‐free cell lines.

### 2.2 Establishment of stable melanoma cell lines

DP‐0574 and IM‐0223 cells (5 × 10^3^ cells/well in 24‐well plates) were transduced with the *ILF2*‐cDNA vector or the empty vector using lentivirus particles (GeneCopoeia, Rockville) in the presence of 5 μg/ml polybrene. M‐204 and IM‐0223 cells (5 × 10^3^ cells/well in 24‐well plates) were transduced with the *ILF2*‐shRNA vector or the empty vector using lentivirus particles (Dharmacon, Colorado) in the presence of 5 μg/ml polybrene. Positive cell lines were selected using Puromycin (Life Technologies, Grand Island). In all the cell lines, ILF2 protein expression was confirmed by western blot. All experiments that involved these cell lines were performed within ten passages after their establishment.

### 2.3 Small interference RNA

M‐204 and IM‐0223 cells (3 × 10^5^ cell/well) were transfected with 25 nM ON‐TARGET plus SMART pool siRNA to downregulate human *U2AF2* or non‐targeting pool siRNA as a control (Dharmacon, Colorado) using jetPRIME (VWR International, Radnor). M‐204 and DP‐0574 cells (3 × 10^5^ cell/well) were transfected with 25 nM ON‐TARGET plus SMART pool siRNA to downregulate human *RAD50* or non‐targeting pool siRNA as a control using jetPRIME. Gene expression was validated 48 h after transfection by western blot.

### 2.4 Cell viability and colony formation assays

The relative cell proliferation was calculated by measuring the number of viable cells at the indicated time points. Cells (2 × 10^3^ cells/well) were cultured in a 96‐well plate (Thermo Fisher Scientific, Waltham), and the number of viable cells was assessed every 24 h using the Cell Titer‐Glo Luminescent Cell Viability assay by the GloMax‐Multi Detection System (Promega, Madison, WI, USA) according to the manufacturers’ instructions.^25^ All the count values obtained at the designated times were relativised to day 1 (time 0). For the colony formation assay, cells (2 × 10^3^ cells/well) were seeded in a 6‐well plate. After 7–10 days of incubation, colonies were then fixed with 100% methanol, stained with 0.3% crystal violet solution and counted using ImageJ software.

### 2.5 Drug treatment

Temozolomide (TMZ, Selleck Chemicals, Houston) was dissolved in dimethyl sulfoxide (DMSO) at a concentration of 200 mM. For melanoma cells, it has been reported that the IC50 concentrations of TMZ ranged from 250 to 800 μM,^26^, ^27^, ^28^, ^29^, ^30^ which was validated in IM‐0223 and DP‐0574 cell lines (Figure S5A–D). For the time‐lapse assay, cells were treated with 600 μM temozolomide, and protein was extracted at 0, 1, 6, 12 and 24 h. For cell viability assays, the measurements were performed after treatment with different concentrations (0, 100, 200, 400, 600 and 800 μM) of TMZ for 72 h. Medium containing only DMSO was used as a negative control and the final volume of DMSO did not exceed 0.4%. For 3D culture, cells were cultured using the 3D spheroid microplates (Corning Inc., New York). Four thousand cells were suspended in 100 μl of medium and then dispensed into the microplates following the manufacturer's instructions. For treatment, a medium containing 600 μM TMZ was replaced on day 4. Spheroids were cultured for 13 days and photographed under the microscope on day 1 and then every 2 days as described previously.^31^, ^32^ ATM kinase inhibitor KU‐55933 (Selleck Chemicals) was dissolved in DMSO at a concentration of 10 mM.

### 2.6 Immunohistochemistry for FFPE tissue

All of the FFPE tissues analysed were provided by SJHC. The cohort consists of 80 FFPE tissues (primary melanomas (*n* = 22), metastatic stage III (*n* = 12) and metastatic stage IV (*n* = 46)) from melanoma patients. FFPE tissues from patients with nevus (*n* = 7) were collected as the normal control. The clinical information for the melanoma patients is described in Table S1. IHC was performed as previously described,^25^, ^33^ using the mouse anti‐human ILF2 Ab (1:250 dilution, Santa Cruz, Cat# sc‐365068) and U2AF2 Ab (1:200 dilution, Santa Cruz, Cat# sc‐53942). Images were taken by the BX43 upright microscope (Olympus, Tokyo) at 20× magnification and with the Mantra Snap Software 1.03 (Perkin Elmer, Waltham). The images were analysed using inForm 2.4 software (Perkin Elmer). H‐scores were calculated following the inForm software instructions available at https://www.perkinelmer.com/ Content/LST_Software_ Downloads/inFormUserManual_2_3_0_rev1.pdf.

### 2.7 RNA in situ hybridisation

All of the FFPE tissues analysed were obtained from the SJHC pathology department. The cohort consists of 55 FFPE tissues [primary melanomas (*n* = 18), metastatic stage III (*n* = 17) and metastatic stage IV (*n* = 20)] from melanoma patients. FFPE tissues from the nevus (*n* = 12) were also collected as the normal control. The clinical information for those patients is described in Table S1. RNA ISH assays were processed according to the manufacturer's instructions and as previously reported.^34^ Tissue slides (5 μm) were stained with the Hs‐ILF2 RNA probe and/or U2AF2 RNA probe (Advanced Cell Diagnostics, Newark) using RNAscope Multiplex Fluorescent Kit V2 according to the manufacturer's instructions available at https://acdbio.com/technical‐support/user‐manuals. Images were taken using an Olympus BX43 upright microscope with 20× magnification and analysed by inForm 2.4 software to calculate the number of foci per cell.

### 2.8 Immunofluorescence staining

For quantification of the number of nucleus per cell, M‐204 and IM‐0223 cells were seeded on Falcon culture slides (Corning), fixed by 4% paraformaldehyde (Thermo Fisher Scientific) for 15 min, rinsed with 100 mM Glycine (Sigma), permeabilised with 0.1% Triton X‐100 for 10 min, and then blocked with 5% bovine serum albumin for 30 min. The cells were then stained with Texas Red‐X Phalloidin (1:100, Thermo Fisher Scientific) for 30 min at room temperature. Nuclei were stained with DAPI. Coverslips were mounted using ProLong Gold antifade. Images were taken by the BX43 upright microscope at 40× magnification and with the Mantra Snap Software 1.03.

### 2.9 Confocal microscopy

For ILF2 and U2AF2 co‐localisation, M‐204 and DP‐0574 cells were treated with TMZ and then processed as explained in the Immunofluorescence staining section. Cells were then stained with rabbit anti‐human ILF2 Ab (1:100, Abcam) and mouse anti‐human U2AF2 Ab (1:100, Santa Cruz). Then Cy™3 goat anti‐rabbit IgG (1:600, Jackson ImmunoResearch, West Grove, PA) and Alexa Fluor 488 goat anti‐mouse IgG (1:600, Jackson ImmunoResearch) were used as the secondary Abs. For quantification of γ‐H2AX foci, DP‐0574 ILF2‐OV and EV cells were stained with rabbit anti‐human γ‐H2AX primary Ab (1:50, Novus Biologicals, Littleton, CO) and Cy™3 goat anti‐rabbit IgG secondary Ab (1:600). In all the experiments, nuclei were stained with DAPI. Coverslips were mounted using ProLong Gold antifade. Confocal images were taken on a Leica TCS‐SP8 inverted spectral confocal microscope (Leica Microsystems: Mannheim, Germany) equipped with a 405 nm blue diode laser, argon laser (5 lines), and white light laser for excitation. Images were processed using Leica software and merge using ImageJ software (http://imagej.nih.gov/ij/).

### 2.10 Co‐immunoprecipitation (Co‐IP) assays

Three melanoma cell lines were washed with PBS and lysed in the immunoprecipitation buffer [150 mM NaCl, 100 mM Tris‐HCl (pH 8), 1% NP‐40, protease and phosphatase inhibitors] by gently pipetting. Protein A‐magnetic beads (Thermo Fisher Scientific) were incubated with 5 μg of rabbit anti‐ILF2 IgG or 5 μg rabbit control IgG for 2 h at 4°C in a rotator. In all of the conditions, the beads were washed three times with the washing buffer [150 mM NaCl, 100 mM Tris‐HCl (pH 8)] on a magnetic rack, and then incubated overnight with 250 μg of whole cell lysate at 4°C with a rotator. Beads were washed three times with the immunoprecipitation buffer and then boiled in the protein loading buffer for 5 min at 95°C in a dry bath. All the samples collected were analysed by western blot. For the reciprocal Co‐IP assay, the protocol was the same except for the following steps. Protein A‐magnetic beads were incubated with 5 μg of rabbit anti‐U2AF2 IgG or 5 μg rabbit control IgG and incubated for 1 h. After the wash step, 250 μg whole cell protein extraction was added and incubated for 1 h. After the wash step, recombinant ILF2‐DDK protein was added and incubated for 1 h. All the samples collected were analysed by western blot.

### 2.11 Nuclear extraction

Nuclear and cytoplasmic fractions were isolated from the DP‐0574 cell line with the Nuclear Extract Kit (Active Motif, Carlsbad). Cells (8.8 × 10^6^ cells/dish) were cultured in 100 mm dishes and harvested with 3 ml cold PBS/Phosphatase inhibitor buffer. Cells were centrifuged and the whole‐cell pellet was gently suspended in 500 μl 1× hypotonic buffer and incubated for 15 min on ice. Then, 25 μl of detergent was added to induce cell lysis. After cell lysis, the cytoplasmic fraction (supernatant) was separated from the nuclear fraction (pellet) by centrifugation (30 s at 14 000 × *g*). Then, the nuclear fraction (pellet) was resuspended in 50 μl of the complete lysis buffer and incubated with 2.5 μl detergent for 30 min on ice. The nuclear lysates were centrifuged for 10 min at 14 000 × *g*. The nuclear fraction (supernatant) was then collected. Both nuclear and cytoplasmic fractions were analysed by western blot.

### 2.12 Western blot assays

Traditional western blot was performed as previously described,^31^, ^32^, ^33^ except for the antibodies utilised that are summarised in Table S2. To visualise the IgG heavy and light chains in Co‐IP assays, membranes were blocked with 3% milk for 30 min and incubated with anti‐rabbit secondary antibody (1:50 000, Cat# 042206, ProteinSimple) for 30 min. All western blot images were analysed with ImageJ software (http://imagej.nih.gov/ij/). All the uncropped western blot images were included in Figures S8–S10.

### 2.13 Quantitative real‐time PCR

RNA was extracted by the ZR‐Duet DNA/RNA MiniPrep kit (Zymo). Turbo DNase (Thermo Fisher) was used to remove DNA contamination. cDNA synthesis was completed by M‐MLV reverse transcriptase (Promega) with Oligo (dt) primer and random primers. The qPCR was performed using PerfecTa SYBR Green SuperMix (Quanta) in LightCycler^®^ 96 System (Roche, Mannheim, Germany). The sequences for all primers utilised are listed in Table S2. Quantitative expression was performed using human SDHA (Succinate Dehydrogenase Complex, Subunit A) as a reference gene and 2^(–ddCT)^ normalisation.^31^

### 2.14 Homologous recombination assay

The HR efficiency in melanoma cells was measured by using the Homologous Recombination Assay Kit (Norgen, Canada). This is a qPCR‐based assay for rapid and qualitative analysis of the HR efficiency between two plasmids (dl‐1 and dl‐2) with different mutations for the lacZa coding region. Briefly, 5 × 10^4^ cells/well were seeded in a 24‐well plate with 500 μl of medium. After 24 h, cells were transfected with 0.5 μg positive control plasmid, 0.5 μg dl‐1 negative plasmid, 0.5 μg dl‐2 negative plasmid, or 1 μg dl assay plasmids mixture (0.5 μg dl‐1 and 0.5 μg dl‐2) respectively, using jetPRIME transfection reagent. After 16 h, cells were collected for DNA isolation by the Quick‐gDNA™ MiniPrep Kit (Zymo, Irvine). The Qubit™ dsDNA BR Assay Kit (Life Technologies, Carlsbad, CA) was used for testing DNA quality and DNA quantification. The assay primer mixtures provided by the manufacturer were used for the qPCR experiment.

### 2.15 Reverse‐phase protein array (RPPA)

Protein lysate was extracted as previously described^31^ from melanoma cell lines (DP‐0574 and IM‐0223) with ILF2‐OV and their respective controls. RPPA analysis was performed by the CCSG‐supported RPPA Core Facility at the University of Texas MD Anderson Cancer Center.^31^ Differences in protein expression between groups were determined using the student's *t*‐test with a two‐sided *p* < .05.

### 2.16 Flow cytometric analysis

For cell cycle assay, cell suspensions of M‐204 sh‐Ctrl and sh‐ILF2 (5 × 10^5^ cells in 0.5 ml complete RPMI medium) were filtered through a nylon mesh (40 μm, BD Falcon) to remove cell clumps. The cells were stained with Vybrant® DyeCycle™ Ruby in 5 μM final concentration (Cat# V10309, Thermo Fisher Scientific) at 37˚C for 30 min in the dark. The cells were analysed using BD FACS Melody (BD Biosciences, Franklin Lakes, NJ) based on the fluorescence emission intensity, which was correlated with the DNA content. Apoptosis was measured using Annexin V‐PE Apoptosis Detection Kit I (Cat# 559763, BD Biosciences). A total of 1 × 10^5^ cells of M‐204 sh‐Ctrl or sh‐ILF2 (in 100 μl binding buffer) were stained with 5 μl Annexin V‐PE and 5 μl 7‐AAD for 15 min at RT in dark. Four hundred microliters of binding buffer was then added to the samples and analysed using BD FACS Melody.

### 2.17 Cell invasion assay

Basement membrane extract cell invasion assay kit (Cat# 3455‐096‐K) was used for cell invasion assay. Briefly, melanoma cells were incubated 24 h in 0.5% FBS heat‐inactivated RPMI medium before harvesting cells (1 × 10^6^ cells/ml in serum‐free RPMI medium). Each top chamber of 96‐plate was coated with 50 μl 0.5× BME solution overnight at 37˚C. A 50 μl cell suspension was added to each top chamber after aspirating off the coating solution. A 150 μl 10% FBS RPMI medium was added to the bottom chamber and incubated at 37˚C for 48 h. The invasion percentages of ILF2‐OV cell lines (DP‐0574 and IM‐0223) were quantified according to the manufacturer's instructions.

### 2.18 Biostatistics

All the statistical analyses were performed using GraphPad Prism 7 software (GraphPad software Inc., La Jolla) or R 3.5.0 version^35^ in a two‐tailed way. The distribution and variation within each group of data were assessed before selecting the correct statistical analysis. Multiple groups were analysed by one‐ or two‐way ANOVA followed by post hoc tests. The correlation was determined by Spearman's or Pearson's correlation test. OS was calculated from the time of the first specimen analysed from the patient until death or last contact. OS was analysed using the Kaplan–Meier method and log‐rank test. All the figures were unified using Adobe Illustrator CC (Adobe Inc., Los Angeles).

## 3 RESULTS

### 3.1 ILF2 expression is associated with melanoma progression

**FIGURE 1:**
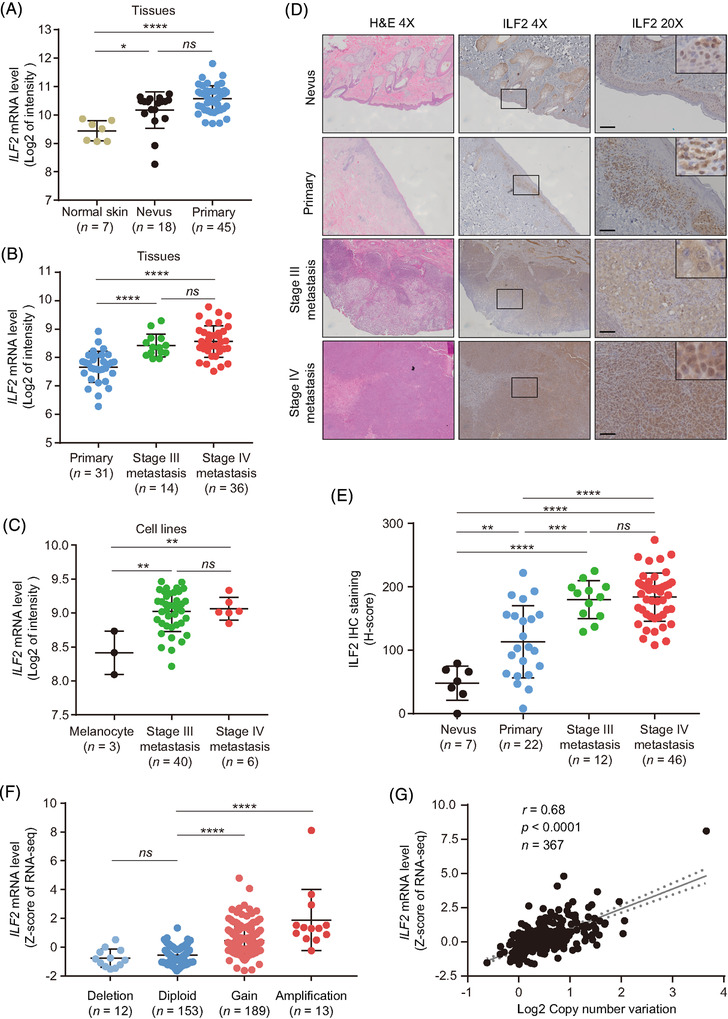
ILF2 is highly expressed in metastatic melanoma. (A) Comparison of *ILF2* mRNA expression in normal skin, nevus and primary melanoma tissues using Talantov microarray dataset. (B) Comparison of *ILF2* mRNA expression in primary, stage III metastasis and stage IV metastasis melanoma tissues using Xu microarray dataset. (C) Comparison of *ILF2* mRNA expression in melanocyte, stage III metastasis and stage IV metastasis melanoma cell lines using SJCI microarray dataset. (D and E) Representative H&E and IHC images (D) and H‐scores quantification (E) of ILF2 in nevus, primary, stage III metastasis and stage IV metastasis melanoma FFPE tissues samples. Scale bars = 50 μm. (F) Comparison of *ILF2* mRNA expression (Z‐score of mRNA expression) in patients with a shallow deletion (Log2 CNV < –0.1), diploid (–0.1 < Log2 CNV < 0.1), gain (0.1 < Log2 CNV < 1.5) and amplification (Log2 CNV > 1.5) on the *ILF2* gene. (G) Correlation between *ILF2* mRNA expression and Log2 CNV using TCGA SKCM dataset. The best‐fit line (straight line) and the 95% confidence intervals (dotted line) were shown in grey. Data represent the mean ± SD. *ns*: not significant, *p < .05, **p < .01, ***p < .001 and ****p < .0001. The correlation was determined by Spearman's (G) correlation test

To examine ILF2 expression in cutaneous melanoma, three different molecular datasets were assessed. The first cohort included a microarray dataset for normal skin, nevus and primary melanoma tissues (GSE3189, *n* = 70).^36^ *ILF2* mRNA average level showed a 2.24 fold‐change enhancement in primary melanomas compared to normal skin tissues (*p *< .0001, Figure 1A). The second melanoma cohort contained primary and metastatic melanoma tumour tissues (GSE8401, *n* = 81).^37^ *ILF2* mRNA average levels were significantly increased in stage III (1.64 fold‐change) and stage IV (1.87 fold‐change) metastatic melanoma compared to primary tumour tissues (Figure 1B). Using the SJCI's microarray dataset, enhanced *ILF2* mRNA levels were also observed in metastatic melanoma cell lines compared to melanocytes (*p *< .01, Figure 1C). To validate these observations, immunohistochemistry (IHC) analysis was performed in nevus, primary and metastatic melanoma (stage III and IV) tissues (Figure 1D). Consistently, the tumour tissues from stage III and IV metastatic melanoma patients had significantly increased ILF2 protein average levels (1.56 and 1.62 fold‐change, respectively) compared to the primary melanoma tissues (Figure 1E). All these observations were consistent with the results of RNA in situ hybridisation (RNA ISH) for *ILF2* mRNA expression in melanoma FFPE tissues (Figure S1A–E).

We then assessed the CNV data from the TCGA SKCM database to determine whether genomic amplifications may be responsible for promoting ILF2 upregulation in metastatic melanoma. Fifty‐five per cent of the metastatic melanoma tumours showed increased copy number of the *ILF2* gene (Figure 1F). Additionally, *ILF2* mRNA expression significantly positive correlated with CNV (*r* = 0.68, *p *< .0001, Figure 1G). We then determined the association between *ILF2* mRNA expression and overall survival (OS). Metastatic melanoma patients with high *ILF2* mRNA expression had shorter OS time compared to patients with low *ILF2* mRNA expression (*p* = .026, Figure S1F). To summarise, ILF2 mRNA and protein expression are increased during cutaneous melanoma progression due to *ILF2* gene amplification. Enhanced *ILF2* mRNA expression in metastatic melanoma patients is significantly associated with a poor prognosis.

### 3.2 ILF2 promotes cell proliferation in metastatic melanoma cell lines

**FIGURE 2:**
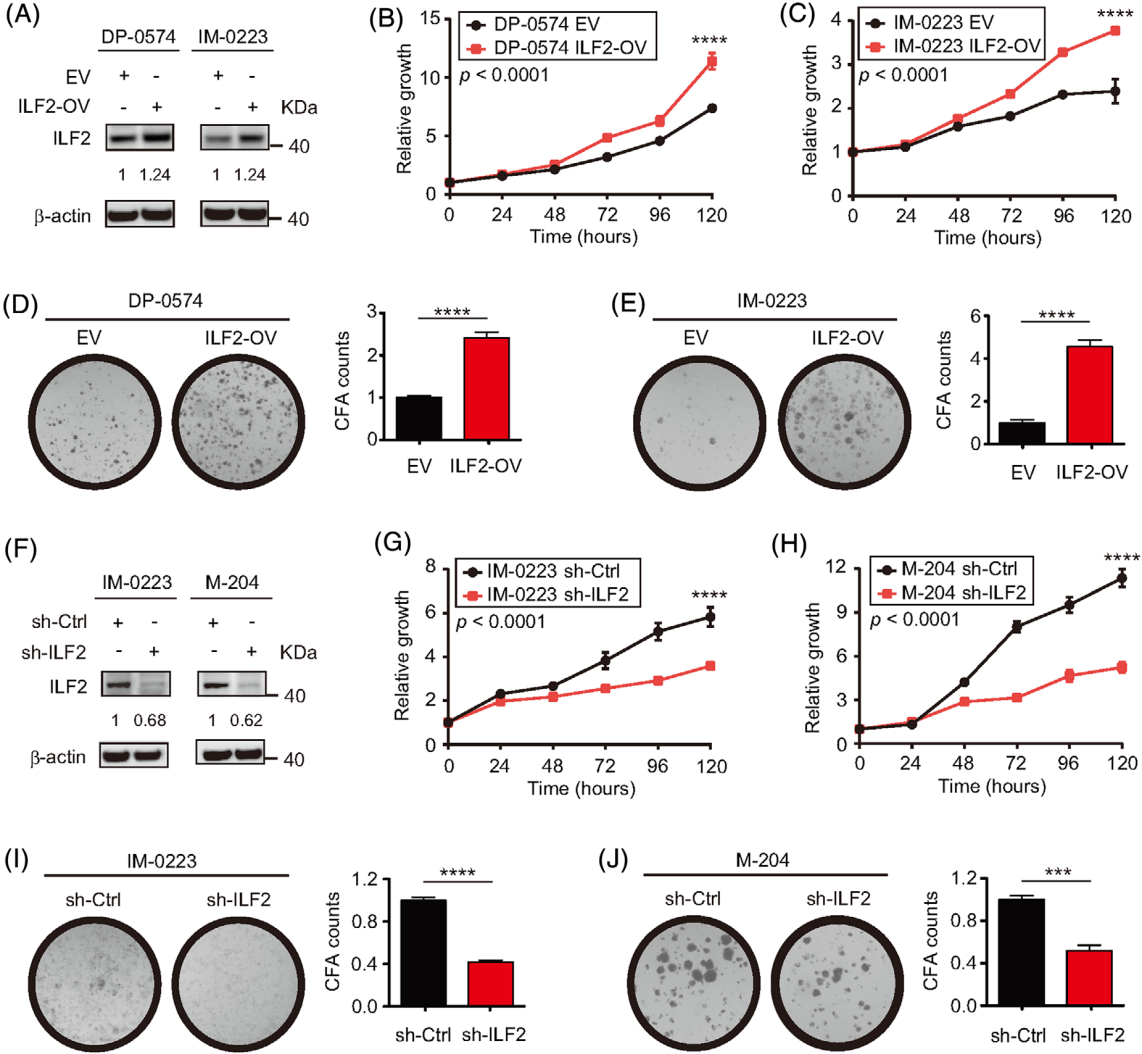
ILF2 expression determines cell proliferation and colony formation ability in metastatic melanoma. (A) Western blot and quantification for ILF2 in two melanoma cell lines with ILF2 overexpression (ILF2‐OV). β‐actin was used as the loading sample control. (B and C) Proliferation assays in two melanoma cell lines with ILF2‐OV. (D and E) Representative colony formation images and quantification plots for the two melanoma cell lines with ILF2‐OV. (F) Western blot and quantification for ILF2 in two melanoma cell lines with ILF2 knockdown. β‐actin was used as the loading sample control. (G and H) Proliferation assays in two melanoma cell lines with ILF2 knockdown. (I and J) Representative colony formation images and quantification plots for the two melanoma cell lines with ILF2 knockdown. Data represent the mean ± SD. ***p < .001 and ****p < .0001

Metastatic melanoma cell lines (DP‐0574 and IM‐0223) were selected to perform ILF2 overexpression (ILF2‐OV) based on the relative expression levels of ILF2 (Figure S1G). ILF2‐OV was confirmed by western blot in DP‐0574 and IM‐0223 cell lines (Figure 2A). Metastatic melanoma cell lines with ILF2‐OV showed higher proliferation rates compared to empty vector (EV) control cell lines (*p *< .001 and *p *< .001, respectively; Figure 2B and C). Similar results were obtained in colony formation assays, where the number of colonies was 2.42 fold‐change in DP‐0574 and 4.56 fold‐change in IM‐0223 (*p *< .001 and *p *< .001, respectively; Figure 2D and E). In invasion assays, melanoma cells with ILF2‐OV showed a higher percentage of invasion (35.6% in DP‐0574 and 26.3% in IM‐0223) compared to control EV cells (8.5% in DP‐0574 and 10.3% in IM‐0223, Figure S1H and I). To determine whether decreased ILF2 expression reduced cell proliferation, we selected two metastatic melanoma cell lines (IM‐0223 and M‐204, Figure S1G) to generate stable knockdown for ILF2. The ILF2 expression was confirmed by western blot in IM‐0223 and M‐204 cell lines (Figure 2F). ILF2 downregulation significantly decreased cell proliferation in IM‐0223 and M‐204 cell lines (*p *< .001 and *p *< .001, respectively; Figure 2G and H). Also, ILF2 downregulation significantly decreased colony formation to 0.42 fold‐change in IM‐0223 and 0.52 fold‐change in M‐204 (*p *< .001 and *p *< .001, respectively; Figure 2I and J). Consistent with a decreased cell proliferation, melanoma cells with ILF2 depletion underwent cell cycle arrest in the G1 phase (*p *< .05, Figure S2A–C) and showed decreased expression for CHK2 (Figure S2D), which is a critical checkpoint factor for cell cycle.^38^ Additionally, ILF2 knockdown significantly induced late apoptosis in melanoma cells (*p *< .001, Figure S2E–G). In summary, ILF2 expression levels significantly correlate with melanoma cell proliferation, colony formation and invasion rates. Moreover, ILF2 depletion promotes cell cycle arrest in the G1 phase and enhances apoptosis in metastatic melanoma cells.

### 3.3 U2AF2 expression is associated with ILF2 expression in melanoma

**FIGURE 3:**
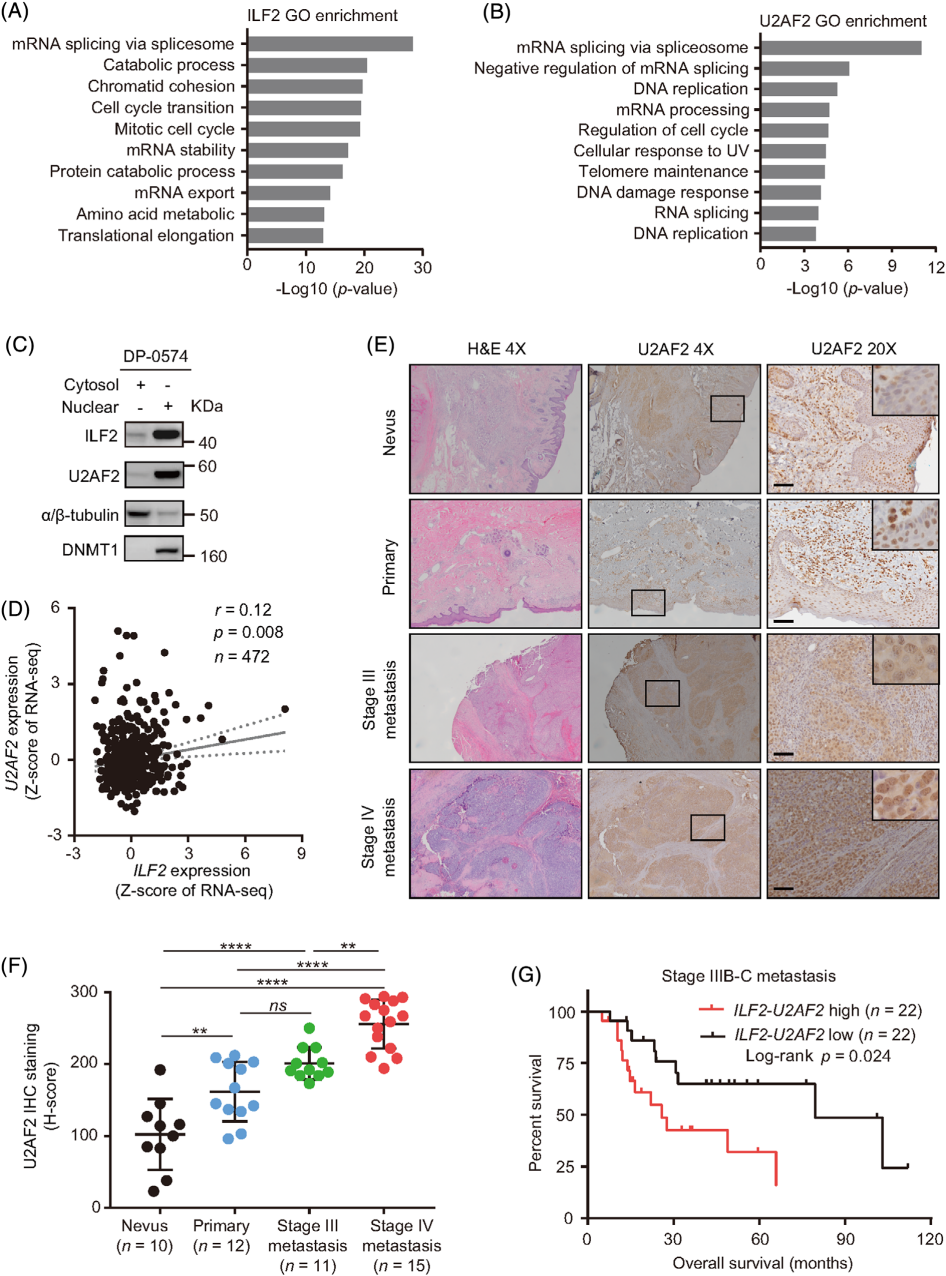
ILF2 is functionally associated with U2AF2 in metastatic melanoma. (A) Gene Ontology (GO) enrichment for ILF2 biological processes based on co‐expression networks from the Coexpedia platform. The biological processes were ranked by –Log10 (*p* value). (B) The top 10 biological processes of U2AF2 GO enrichment from the Coexpedia platform. (C) Western blot of ILF2 and U2AF2 proteins in the cytosol and nuclear fractions of DP‐0574 melanoma cell lines. DNMT1 was used as the loading control for the nuclear fractions and α/β‐tubulin was used as the loading control for the cytosol fractions. (D) Correlation between *ILF2* and *U2AF2* mRNA expression obtained from the TCGA SKCM RNA‐seq dataset. (E and F) Representative H&E and IHC images (E) and H‐score quantitation (F) of U2AF2 in nevus, primary melanoma, stage III metastasis and stage IV metastasis melanoma FFPE samples. Scale bars = 50 μm. (G) OS curve for TCGA SKCM stage IIIB–C metastatic patients that were divided according to *ILF2* and *U2AF2* mRNA expression levels into lower and upper quartile. *ns*: not significant, **p < .01 and ****p < .0001

In two previous studies, the interaction of ILF2 and U2AF2 has been demonstrated.^16^, ^39^ Marchesini et al. showed that ILF2 is functionally related to the essential splicing factor U2AF2^16^. Meanwhile, Whisenant et al. found that ILF2 binds to U2AF2 and regulates gene expression in human CD4 T cells.^39^ As a component of the ribonucleoprotein complex, U2AF2 plays a critical role in pre‐mRNA splicing and 3′‐end processing.^40^, ^41^, ^42^, ^43^ We then focused on the identification of the signalling pathways and biological processes modulated by ILF2. Gene Ontology (GO) analysis for ILF2 biological processes from the Coexpedia platform^44^ showed the enrichment of mRNA‐related metabolic pathways (Figure 3A). We observed similar GO enrichment for U2AF2 biological processes (Figure 3B). Consistently, we observed that both ILF2 and U2AF2 proteins showed nuclear localisation in metastatic melanoma cell lines (Figure 3C). Moreover, *ILF2* and *U2AF2* mRNA levels had a significant positive correlation in melanoma (primary and metastasis) tumour samples from TCGA SKCM dataset (*r* = 0.12, *p *= .008, Figure 3D). Additionally, stage III melanoma patients with high *ILF2* mRNA expression had significantly higher *U2AF2* mRNA levels compared to patients with low *ILF2* mRNA expression (*p *< .05, Figure S3A). A significant upregulation of *U2AF2* mRNA levels was observed in primary melanoma compared to normal skin (*p *< .05) and nevus tissues (*p *< .0001) using GSE3189 microarray dataset (Figure S3B). Also, stage III derived‐melanoma cell lines showed higher *U2AF2* mRNA levels than melanocyte cell lines in the SJCI's microarray dataset (*p *< .05, Figure S3C). These results were further validated using western blot (Figure S3D), RNA ISH (Figure S3E and F) and IHC analysis (Figure 3E and F). Consistently, ILF2 and U2AF2 expression levels showed a significant positive correlation in RNA ISH (*r* = 0.63, *p *< .001) and IHC assays (*r* = 0.47, *p *< .05, Figure S3G and H). Additionally, metastatic melanoma patients (stage IIIB–C) with high *ILF2*‐*U2AF2* mRNA levels showed significantly shorter OS compared to patients with low *ILF2*‐*U2AF2* mRNA levels (*p *= .024, Figure 3G). These results suggested that ILF2 and U2AF2 play a role in promoting melanoma tumour progression to advanced stages.

### 3.4 U2AF2 is required for ILF2 to promote proliferation in melanoma cell lines

**FIGURE 4:**
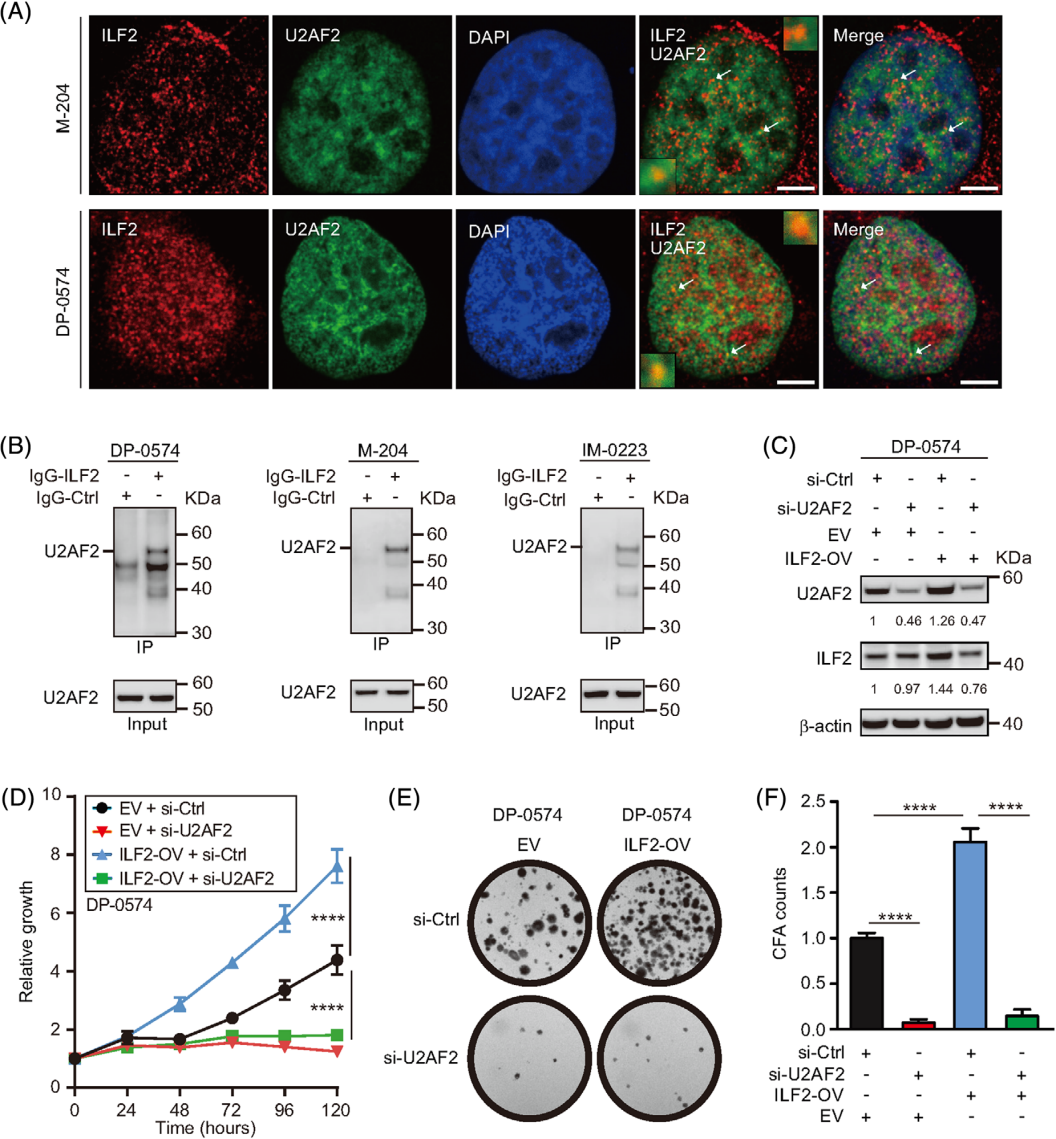
U2AF2 is required for ILF2 to promote melanoma cell proliferation. (A) Confocal images for ILF2 (red) and U2AF2 (green) proteins in M‐204 and DP‐0574 melanoma cells. The nuclei were stained with DAPI (blue). The arrowheads show the co‐localisation of ILF2 and U2AF2. Scale bars = 2 μm. (B) Co‐immunoprecipitation assay for ILF2 and U2AF2 in melanoma cell lines. (C) Western blot and quantification for ILF2 and U2AF2 in melanoma cells with ILF2 overexpression (ILF2‐OV) or control empty vector (EV) transfected with U2AF2 siRNA (si‐U2AF2) or control siRNA (si‐Ctrl). β‐actin was used as the loading sample control. (D) Proliferation assay in melanoma cells with ILF2‐OV or control cells transfected with si‐U2AF2 or si‐Ctrl. (E and F) Representative colony images and quantification in melanoma cells with ILF2‐OV or control empty vector (EV) cells transfected with si‐U2AF2 or si‐Ctrl. ****p < .0001

To determine whether the correlations described above were due to the interaction between ILF2 and U2AF2 proteins, co‐localisation using confocal microscopy and co‐immunoprecipitation assays were performed. The results showed that ILF2 interacts with U2AF2 in the nucleus of metastatic melanoma cell lines (Figures 4A and S4A). Co‐immunoprecipitation assay using the ILF2 antibody showed that U2AF2 binds to ILF2 (Figure 4B). Moreover, reciprocal co‐immunoprecipitation using the U2AF2 antibody demonstrates that ILF2 binds to U2AF2 (Figure S4B and C). Then, we functionally characterised U2AF2 by performing knockdown assays in melanoma cell lines. U2AF2 knockdown was confirmed by western blot (Figure S4D). U2AF2 knockdown significantly decreased cell proliferation (*p *< .0001) and colony formation (*p *< .0001) compared to the respective control cell lines (Figure S4E–H). Furthermore, U2AF2 was depleted in ILF2‐OV or control cell lines to determine the importance of U2AF2 in driving ILF2‐induced effects. U2AF2 knockdown blocked the enhanced cell proliferation (*p *< .0001) and colony formation (*p *< .0001) induced by ILF2‐OV in metastatic melanoma (Figure 4C–F). In summary, ILF2 forms a complex with U2AF2 to promote cell proliferation and colony formation in metastatic melanoma.

### 3.5 ILF2 controls DNA damage response in metastatic melanoma cells

**FIGURE 5:**
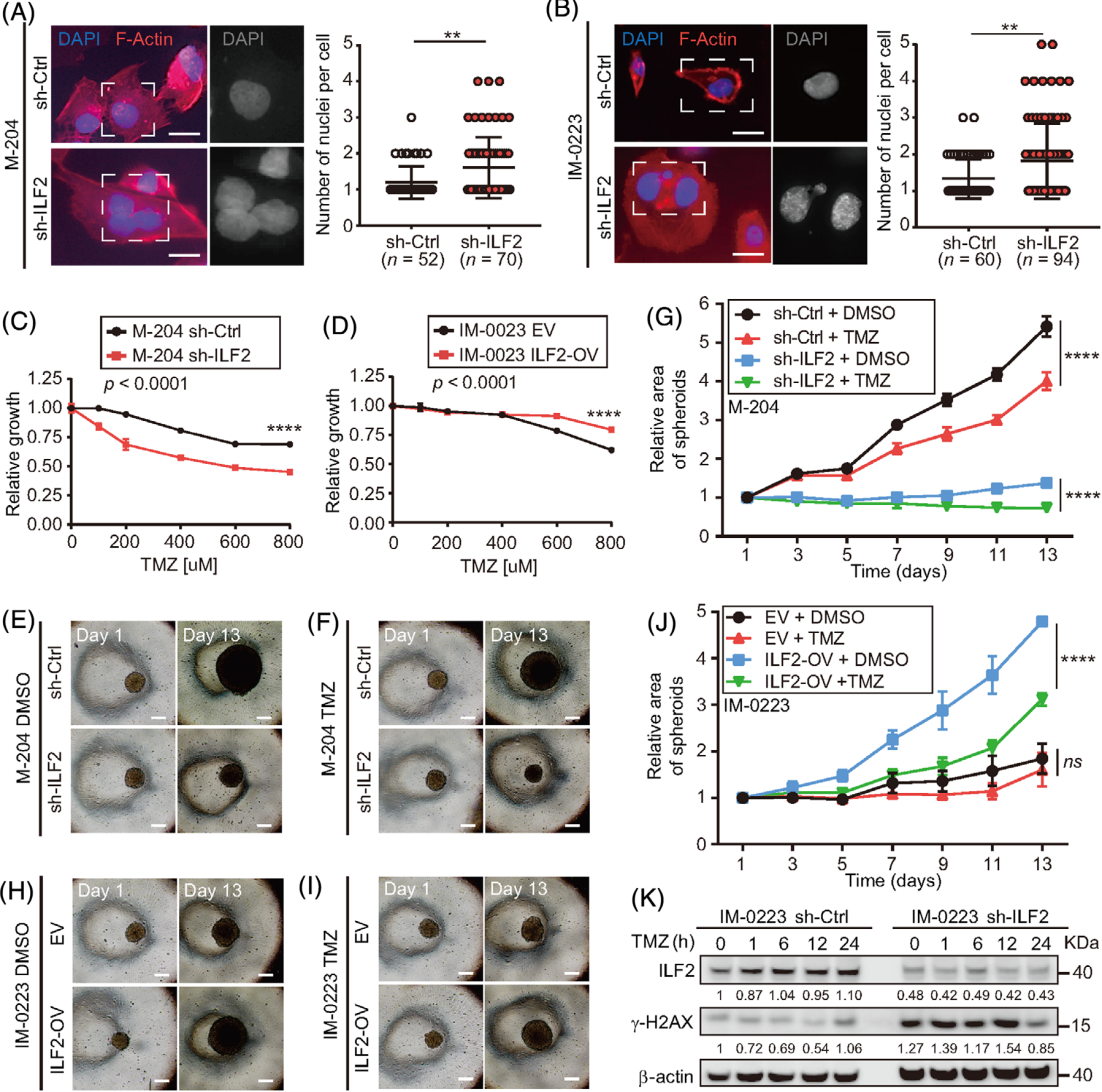
ILF2 controls DNA damage response in metastatic melanoma cell lines. (A and B) Representative images and quantification of the number of nuclei per cell in M‐204 (A) and IM‐0223 (B) cells transduced with the ILF2 shRNA (sh‐ILF2) or non‐silencing shRNA (sh‐Ctrl). The nuclei were stained with DAPI (blue) and F‐actin was stained with Texas‐Red‐X phalloidin (red). Scale bar = 10 μm. (C and D) Drug sensitivity assays for melanoma cell lines with sh‐ILF2 (C) or ILF2‐OV (D) that were treated with different concentrations of TMZ for 3 days and compared to their respective controls. (E–J) Representative images at day 1 and day 13 (E, F, H and I) and quantification of spheroids area (G, J) of melanoma cell lines with ILF2 knockdown (sh‐ILF2) or ILF2 overexpression (ILF2‐OV) treated with 600 μM TMZ for 24 h starting at day 4. Scale bar = 50 μm. (K) Western blot and the quantification of ILF2 and γ‐H2AX in melanoma cells treated with 600 μM TMZ for the indicated time (1, 6, 12 and 24 h). β‐actin was used as the loading sample control. Data represent the mean ± SD. *ns*: not significant, **p < .01 and ****p < .0001

Previous studies demonstrated the potential link between reduced ILF2 expression and decreased DNA damage repair (DDR) in multiple myeloma and gastric cancer.^16^, ^45^ Moreover, the decrease in DDR promotes genomic instability and increases the number of multinucleated cells.^46^, ^47^ Consistently, ILF2 knockdown significantly increased the number of nuclei observed per cell in metastatic melanoma cell lines (*p *< .01, Figure 5A and B). Therefore, we hypothesised that ILF2 overexpression may promote enhanced DDR in metastatic melanoma. To address this hypothesis, metastatic melanoma cells were exposed to TMZ (Figure S5A–D), an alkylating chemotherapeutic drug used for the treatment of patients with melanoma brain metastasis.^48^, ^49^ In cell viability assays, metastatic melanoma cell lines with ILF2 knockdown had increased sensitivity to TMZ (*p *< .0001, Figure 5C), while metastatic melanoma cell lines with ILF2‐OV showed increased resistance to TMZ (*p *< .0001, Figure 5D). In 3D spheroids assays, ILF2 knockdown cells exhibited reduced growth by forming significantly (*p *< .0001) smaller spheroids compared to the control cells (Figure 5E), consistent with the results shown in Figure 2. When treated with TMZ, the spheroids formed by ILF2 knockdown and control cell lines showed a significantly smaller spheroid compared to the respective control cell lines without treatment (Figure 5E–G). Most importantly, control cells treated with TMZ formed significantly larger spheroids than ILF2 knockdown cells treated with TMZ (Figure 5G). These results were further validated in melanoma cell lines with ILF2‐OV (Figure 5H–J). In summary, ILF2 knockdown reduces DDR and increases cell sensitivity to TMZ. On contrary, ILF2‐OV enhances DDR and promotes TMZ resistance.

Subsequently, the phosphorylation levels of γ‐H2AX were evaluated to measure the accumulation of double‐strand breaks (DSB) in metastatic melanoma cells treated with TMZ. Enhanced phosphorylation levels of γ‐H2AX were observed in ILF2 knockdown cells compared to control cells, in both TMZ‐treated and untreated conditions (Figures 5K and S5E–H). To summarise, melanoma cell lines with reduced ILF2 expression treated with TMZ are more sensitive to DNA damage and display enhanced accumulation of DSB. In contrast, ILF2‐OV promotes TMZ resistance by reducing DNA damage and the accumulation of DSB. These results reinforced the hypothesis that ILF2 expression is associated with DDR in metastatic melanoma.

### 3.6 Enhanced ILF2 promotes RAD50 expression in metastatic melanoma cells

**FIGURE 6:**
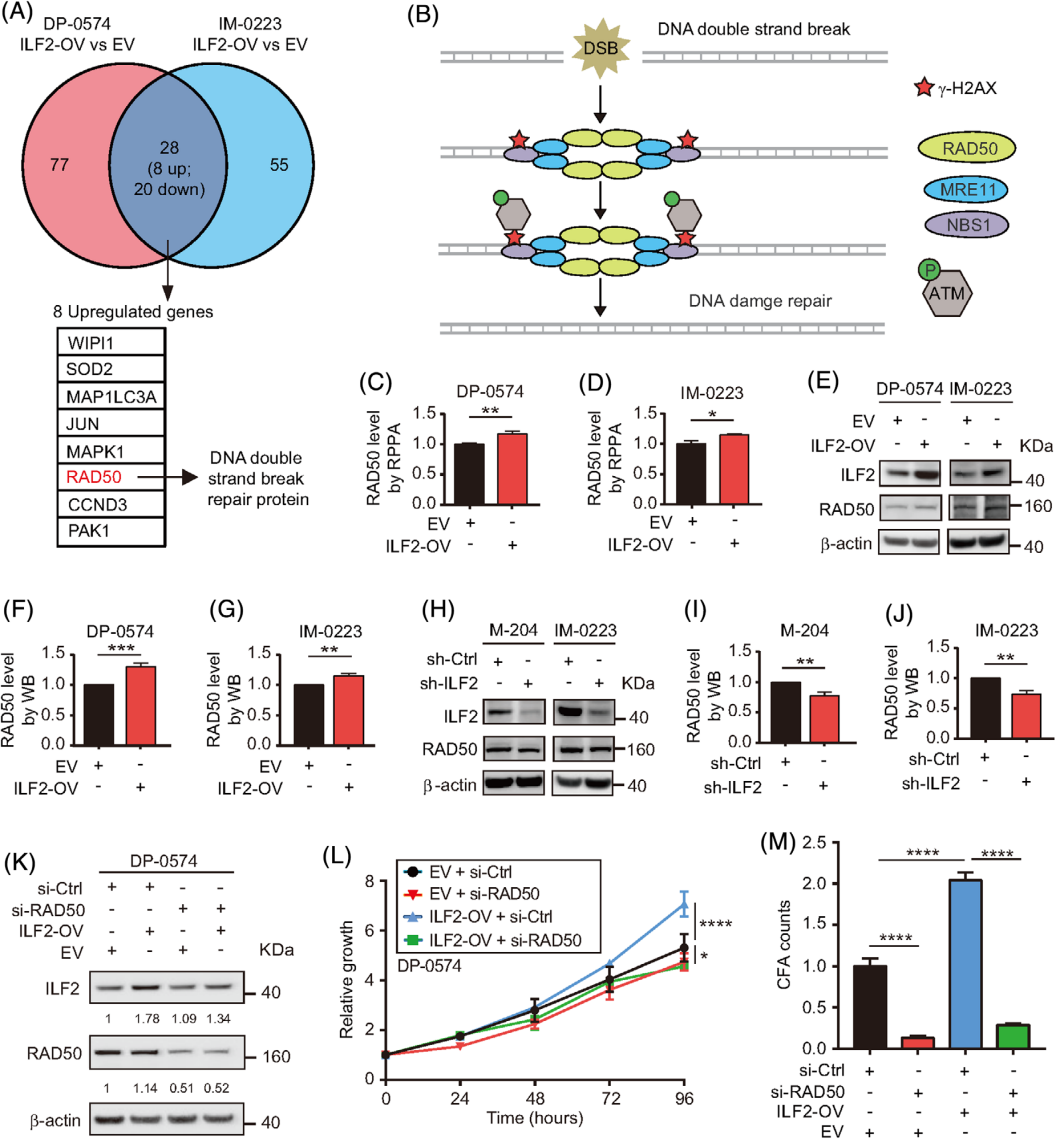
ILF2 overexpression enhances RAD50 expression in melanoma cell lines. (A) Eight commonly and significantly upregulated genes (fold‐change > 1.1 and *p *< .01) were identified by RPPA assays in two ILF2‐OV melanoma cell lines. The DNA damage‐related gene is labelled in red. (B) Schematic diagram showing the role of RAD50 in response to DNA double‐strand break. (C and D) Comparison of RAD50 protein expression in two melanoma cells transduced with ILF2‐OV or EV vector using RPPA dataset. (E–J) Western blot and the quantification of ILF2 and RAD50 in melanoma cells with ILF2‐OV (E–G) or ILF2 knockdown (H–J). (K) Western blot and quantification of ILF2 and RAD50 in ILF2‐OV or EV melanoma cells transfected with si‐RAD50 or si‐Ctrl. (L) Proliferation assay in melanoma cells with ILF2‐OV or EV transfected with si‐RAD50 or si‐Ctrl. (M) Quantification of colony formation in melanoma cells with ILF2‐OV or EV transfected with si‐RAD50 or si‐Ctrl. β‐actin was used as the loading sample control. Data represent the mean ± SD. *p < .05, **p < .01, ***p < .001 and ****p < .0001

To identify the downstream pathway controlled by ILF2 that increases DDR, we compared the two metastatic melanoma cell lines with ILF2‐OV to their respective control cell lines using RPPA analysis. Only eight proteins were significantly upregulated in both cell lines (Figure 6A). Based on these observations, we focused on RAD50 because of its critical role in DDR as a part of the MRE11‐RAD50‐NBS1 (MRN) complex, which interacts with γ‐H2AX to activate ATM‐dependent DNA repair pathways, such as homologous recombination (Figure 6B).^50^, ^51^, ^52^, ^53^ The results from RPPA analysis suggested that ILF2 enhanced DDR by upregulating RAD50 (*p *< .01 in DP‐0574 and *p *< .05 in IM‐0223, Figure 6C and D). The RPPA results were validated by assessing RAD50 protein levels in ILF2‐OV and ILF2 knockdown cell lines using western blot. ILF2‐OV significantly enhanced RAD50 (*p *< .001 in DP‐0574 and *p *< .01 in IM‐0223, Figure 6E–G), while ILF2 knockdown reduced RAD50 (*p *< .01 in M‐204 and *p *< .01 in IM‐0223, Figure 6H–J). Then, RAD50 knockdown was evaluated to determine whether RAD50 plays a role in metastatic melanoma cell proliferation. Consistently, RAD50 knockdown significantly decreased melanoma cell proliferation and colony formation compared to respective control cell lines (Figure S6A–E). Moreover, RAD50 knockdown blocked the enhanced cell proliferation induced by ILF2‐OV in metastatic melanoma (Figure 6K–M). In conclusion, RAD50 plays a significant role as a downstream effector of ILF2, and it may have implications in controlling DDR in metastatic melanoma cells.

### 3.7 Enhanced ILF2‐U2AF2 complex activates the RAD50‐downstream ATM pathway

**FIGURE 7:**
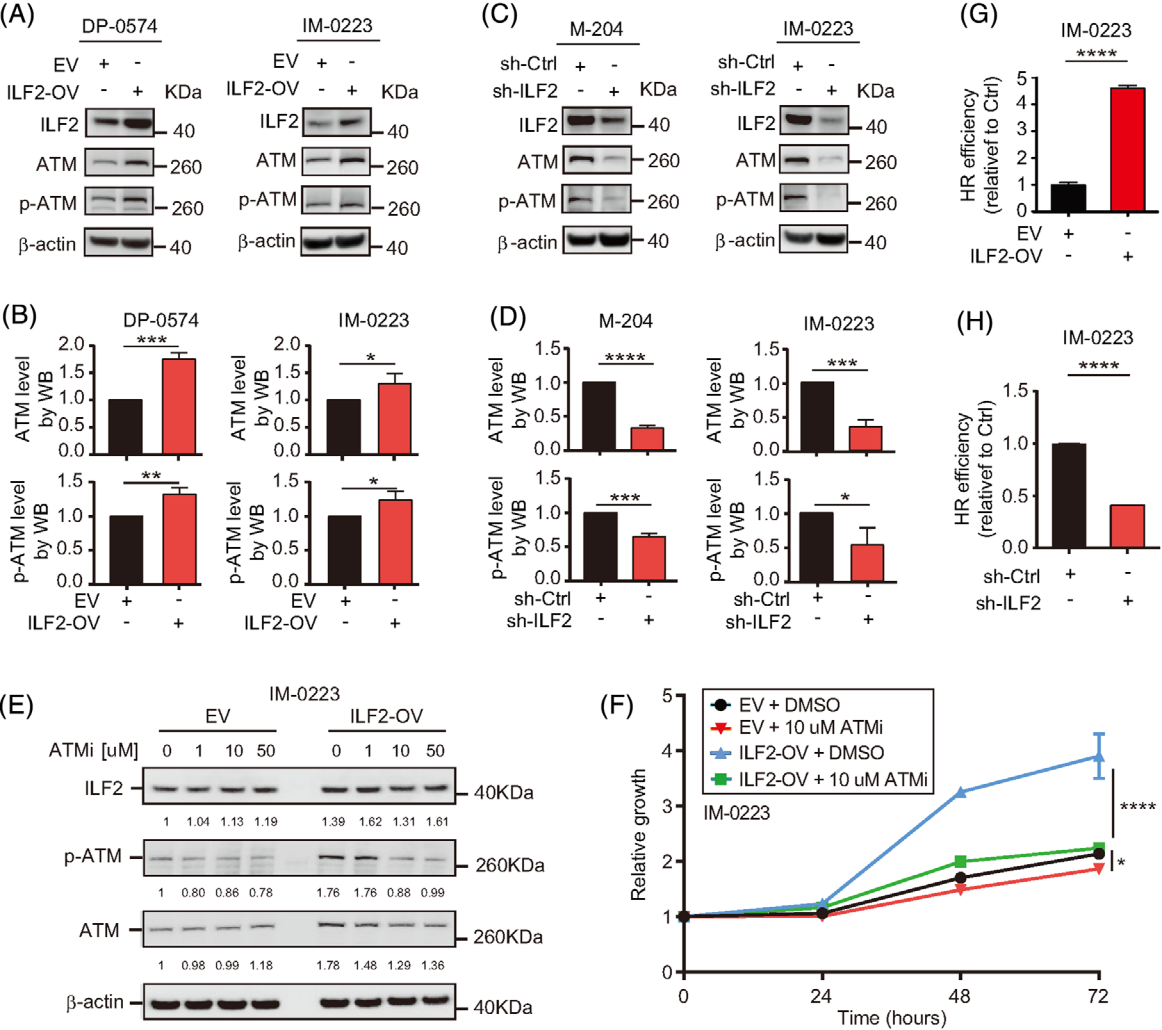
ILF2 mediates the activation of the ATM pathway. (A–D) Western blot and the quantification of ILF2, ATM and p‐ATM in melanoma cell lines with ILF2‐OV (A and B) or ILF2 knockdown (C and D). (E) Western blot and the quantification of ILF2, p‐ATM and ATM in IM‐0223 EV or ILF2‐OV cells treated with DMSO, 1 μM, 10 μM, or 50 μM ATMi for 3 h. (F) Proliferation assay on IM‐0223 melanoma cell lines with EV or ILF2‐OV after treatment with DMSO or 10 μM ATMi for 3 h. β‐actin was used as the loading sample control. (G and H) Homologous recombination efficiency assay on melanoma cell lines with ILF2‐OV (G) or ILF2 knockdown (H). Data represent the mean ± SD. *p < .05, **p < .01, ***p < .001 and ****p < .0001

Based on the results shown above, we inferred that RAD50 upregulation may improve the ATM pathway activation in metastatic melanoma cell lines during DNA damage. To further characterise the role of ILF2, we evaluated the protein and activation levels of ATM, which is a downstream effector of the MRN complex following DNA damage (Figure 6B).^54^ Accordingly, ATM levels were increased in ILF2‐OV cell lines (*p *< .001 in DP‐0574 and *p *< .05 in IM‐0223), and consequently, we observed significant changes in the levels of phosphorylated ATM (p‐ATM) (*p *< .01 in DP‐0574 and *p *< .05 in IM‐0223, Figure 7A and B). Conversely, ATM and p‐ATM levels were consistently and significantly decreased in cell lines with ILF2 knockdown (Figure 7C and D). To explain the changes in ATM protein expression, we analysed the mRNA levels of ATM. Melanoma cells with ILF2‐OV showed a significant upregulation in ATM mRNA levels (Figure S6F). On the contrary, ILF2 knockdown cells significantly reduced ATM mRNA levels (Figure S6G).

Then, we investigated whether the levels of ATM determine the response to ATMi KU‐55933. ILF2‐OV melanoma cells treated with the ATMi KU‐55933 showed reduced cell proliferation than the respective control EV cell lines (Figures 7E and F, S6H and I). Using RNA‐Seq data from the TCGA SKCM database, *RAD50*, *NBS1*, *MRE11* and *ATM* expression were analysed in melanoma tissues. Accordingly, patients with metastatic stage III melanoma tumours had a significantly higher mRNA expression for *RAD50*, *NBS1*, *MRE11* and *ATM* as compared to patients with primary melanoma (Figure S7A–D). Because of the dependence of homologous recombination (HR) on ATM,^55^, ^56^ we investigated the efficiency of HR in ILF2‐OV and ILF2 knockdown cell lines. In agreement with higher RAD50 and ATM protein levels, cell lines with ILF2‐OV had an enhanced efficiency of HR compared to the control cell lines (Figure 7G). On the contrary, significantly lower efficiency of HR was observed in cell lines with ILF2 knockdown (Figure 7H). Our results demonstrated the regulatory role of ILF2 controlling the expression of RAD50 and ATM pathway activation, which consequently enhanced the efficiency of DDR by activating HR in metastatic melanoma cells.

**FIGURE 8:**
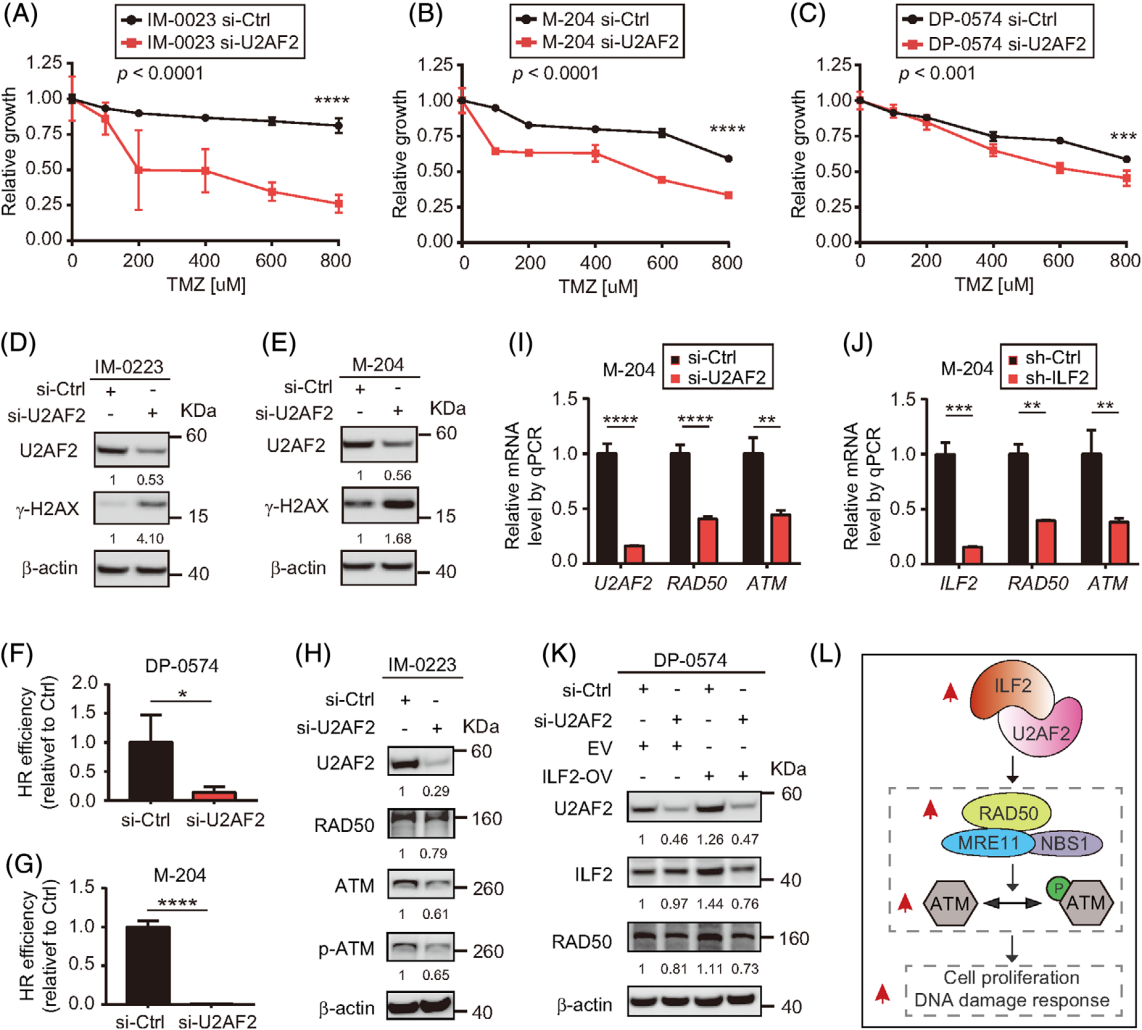
ILF2 regulates the ATM pathway by recruiting U2AF2. (A‐C) Drug sensitivity assays in U2AF2 knockdown melanoma cell lines treated with different concentrations of TMZ for 72 h. (D and E) Western blot and the quantification of U2AF2 and γ‐H2AX in IM‐0223 (D) and M‐204 (E) melanoma cells with U2AF2 knockdown. (F and G) Homologous recombination efficiency assay on U2AF2 knockdown DP‐0574 (F) and M‐204 (G) melanoma cells. (H) Western blot and the quantification of U2AF2, RAD50, ATM and p‐ATM in U2AF2 knockdown cells. (I and J) The RT‐qPCR assay analysing the mRNA expression of *U2AF2*, *RAD50* and *ATM* in melanoma cells transfected with si‐Ctrl or si‐U2AF2 (I), or *ILF2*, *RAD50* and *ATM* in melanoma cells transfected with sh‐Ctrl or sh‐ILF2 (J). (K) Western blot and the quantification of U2AF2, ILF2 and RAD50 in melanoma cells. β‐actin was used as the loading sample control. (L) Proposed schematic model for the function of ILF2 in regulating the ATM pathway in metastatic melanoma. Data represent the mean ± SD. *p < .05, **p < .01, ***p < .001 and ****p < .0001

To validate the function of U2AF2 in regulating the DNA damage response, melanoma cell lines with U2AF2 knockdown were treated with TMZ. As expected, U2AF2 knockdown in melanoma lines led to an increase in sensitivity to TMZ (Figure 8A–C), increased γ‐H2AX levels (Figure 8D and E) and decreased HR efficiency (Figure 8F and G). Also, U2AF2 knockdown significantly reduced the protein levels of RAD50 and ATM (Figure 8H). Moreover, U2AF2 knockdown downregulated *RAD50* and *ATM* mRNA levels (Figure 8I), which is consistent with the decreased *RAD50* and *ATM* mRNA levels observed in cells with ILF2 knockdown (Figure 8J). To demonstrate that the ILF2‐induced effects on the ATM pathway were mediated by ILF2‐U2AF2 interaction, U2AF2 was depleted in ILF2‐OV melanoma cell lines. While ILF2‐OV induced the upregulation of RAD50, U2AF2 knockdown decreased the RAD50 protein levels in ILF2‐OV melanoma cells (Figure 8K). In summary, high levels of ILF2‐U2AF2 protein complex control melanoma progression by upregulating the mRNA and protein expression of RAD50 and ATM (Figure 8L). Consequently, RAD50 and ATM improve DDR and promote resistance to TMZ in metastatic melanoma.

## 4 DISCUSSION

The survival outcomes for cutaneous melanoma have significantly improved due to immune checkpoint inhibitor therapies; however, only a limited number of patients exhibit a durable complete response to treatment over 5 years.^4^, ^57^, ^58^ Thus, distant organ metastatic melanoma management remains a major clinical problem. In this study, we found that 1q21.3 amplification‐driven ILF2 upregulation is associated with melanoma progression. Briefly, ILF2 protein expression was enhanced in metastatic melanoma tumours coupled with increased *ILF2* mRNA expression and gene copy number. Enhanced *ILF2* mRNA expression was associated with poor outcomes in metastatic melanoma patients. Functional assays revealed a regulatory role for ILF2 in promoting cell proliferation and colony formation, which further raises the question of what downstream factors are regulated by ILF2 to promote melanoma tumour progression.

The GO enrichment analysis of ILF2 function suggested the potential relationship with the splicing factor U2AF2. U2AF2 is a subunit of the U2AF complex, which recruits U2 snRNP for spliceosome assembly and plays a critical role in pre‐mRNA editing.^59^, ^60^ Mass spectrometry was used to identify protein interacting partners of U2AF2 upon TCR activation in primary human CD4 T cells.^39^ The authors found several interacting proteins, including ILF2^39^. In addition, ILF2 had previously been implicated in post‐transcriptional regulation of genes related to cytokine secretion.^39^ In another study, ILF2 was found to modulate YB‐1 nuclear localisation in multiple myeloma and promote U2AF2 recruitment dependent on YB‐1.^16^ However, in cutaneous melanoma, little is known about the relationship between ILF2 and U2AF2 or the protein effectors that function downstream the pathway. Here, we used confocal and co‐immunoprecipitation to uncover the existence of the ILF2‐U2AF2 complex in metastatic melanoma cells. ILF2‐U2AF2 complex has critical functions in promoting tumour cell proliferation and colony formation ability. Moreover, high mRNA levels of *ILF2* and *U2AF2* were associated with poor outcomes in metastatic melanoma patients.

A previous study has shown the overlap RNA editing in functions for ILF2 and U2AF2 in cell lines.^61^ Another study showed that ILF2/YB‐1/U2AF2 complex promotes DDR‐related mRNA processing that affects the expression of *FANCD2* and *EXO1* in multiple myeloma.^16^ Our results show that ILF2 and U2AF2 may have implications in *RAD50* and *ATM* mRNA processing in metastatic melanoma. Also, our evidence from RPPA and in vitro assays indicated RAD50 and ATM proteins as downstream effectors of the ILF2‐U2AF2 complex in metastatic melanoma cells. RAD50 is a part of the MRN complex that is an important DDR regulator,^62^, ^63^ thus decreased RAD50 protein levels will reduce DDR and may enhance treatment response in vitro. Our results consistently showed that ILF2‐depleted melanoma cells exhibited multinucleated phenotypes and increased γ‐H2AX levels, as a consequence of the accumulation of DSB.^64^, ^65^ More importantly, ILF2 or U2AF2 downregulation increased the sensitivity to the DNA‐damage agent TMZ in metastatic melanoma cells. Higher ILF2/U2AF2 expression and a consequently significantly higher expression of RAD50 were observed in metastatic melanoma tissues compared to primary tissues. Additionally, metastatic melanoma patients (stage IIIB–C) with low *ILF2*‐*U2AF2* mRNA levels showed increased OS compared to patients with high *ILF2*‐*U2AF2* mRNA levels, which may be explained by the reduced RAD50 levels, the increased sensitivity to DNA‐damage agents, and a higher propensity to genomic instability observed in vitro and in clinical specimens. This finding has clinical translational value and suggests ILF2 and U2AF2 as potential biomarkers for OS in stage IIIB–C melanoma patients, who are at higher risk of developing distant organ metastasis.

RAD50 is essential for MRN's function in DDR by affecting the conformational states of the MRN complex, which is dependent on ATP binding and hydrolysis through the RAD50 protein.^62^, ^66^ ATM is recruited to sites of DNA damage by MRN complex,^54^, ^67^ resulting in ATM activation that plays a critical role in DNA damage response, cell proliferation and apoptosis.^68^, ^69^, ^70^ Along with the elevated RAD50 expression, enhanced ATM and increased p‐ATM levels were observed in metastatic melanoma cells with ILF2‐OV. The upregulated ATM activation observed in ILF2‐OV cells could explain the ILF2‐induced effects on proliferation, resistance to DNA‐damage agents, and high efficiency of HR in metastatic melanoma cells. More importantly, this mechanism may have major implications in driving melanoma progression. Further studies are needed to determine the potential clinical utility of monitoring CNV in ILF2 and other genes located in the 1q21.3 region in pre‐operative blood samples with the aim of monitoring response to the current standard of care treatment.

## 5 CONCLUSIONS

Our results demonstrated that ILF2 expression is associated with 1q21.3 amplification and melanoma progression. These findings help to understand the underlying biology of 1q21.3 amplification that is a frequent event in metastatic melanoma patients.^18^ Molecularly, ILF2 forms a nuclear complex with U2AF2. Enhanced ILF2‐U2AF2 expression promotes cell proliferation and increases RAD50 and ATM mRNA and protein expression. Melanoma cell lines with ILF2 overexpression activate a more effective DNA damage response to TMZ. Therefore, enhanced ILF2‐U2AF2 expression is associated with a shorter time for OS in stage IIIB–C melanoma patients. Interestingly, higher sensitivity to ATMi was observed in melanoma cell lines with ILF2 overexpression.

## CONFLICT OF INTEREST

Gordon B. Mills reports SAB/Consultant: AstraZeneca, Chrysallis Biotechnology, ImmunoMET, Ionis, Lilly, PDX Pharmaceuticals, Signalchem Lifesciences, Symphogen, Tarveda, Zentalis. Stock/Options/Financial: Catena Pharmaceuticals, ImmunoMet, SignalChem, Tarveda Licensed Technology HRD assay to Myriad Genetics, DSP patents with Nanostring Sponsored research Nanostring Center of Excellence, Ionis (Provision of tool compounds). All other authors declare no competing interests.

## Supporting information



Supporting information
