# Supplementary material for: Interleukin enhancer‐binding factor 2 promotes cell proliferation and DNA damage response in metastatic melanoma
Source: Clin Transl Med. 2021 Oct 14;11(10):e608. doi: 10.1002/ctm2.608 (PMC8516365; doi:10.1002/ctm2.608)
Supplement: Supplementary file 1 — Supporting information [file CTM2-11-e608-s001.docx]

**Supplementary information for Figure S1-10 and Table S1-2.**


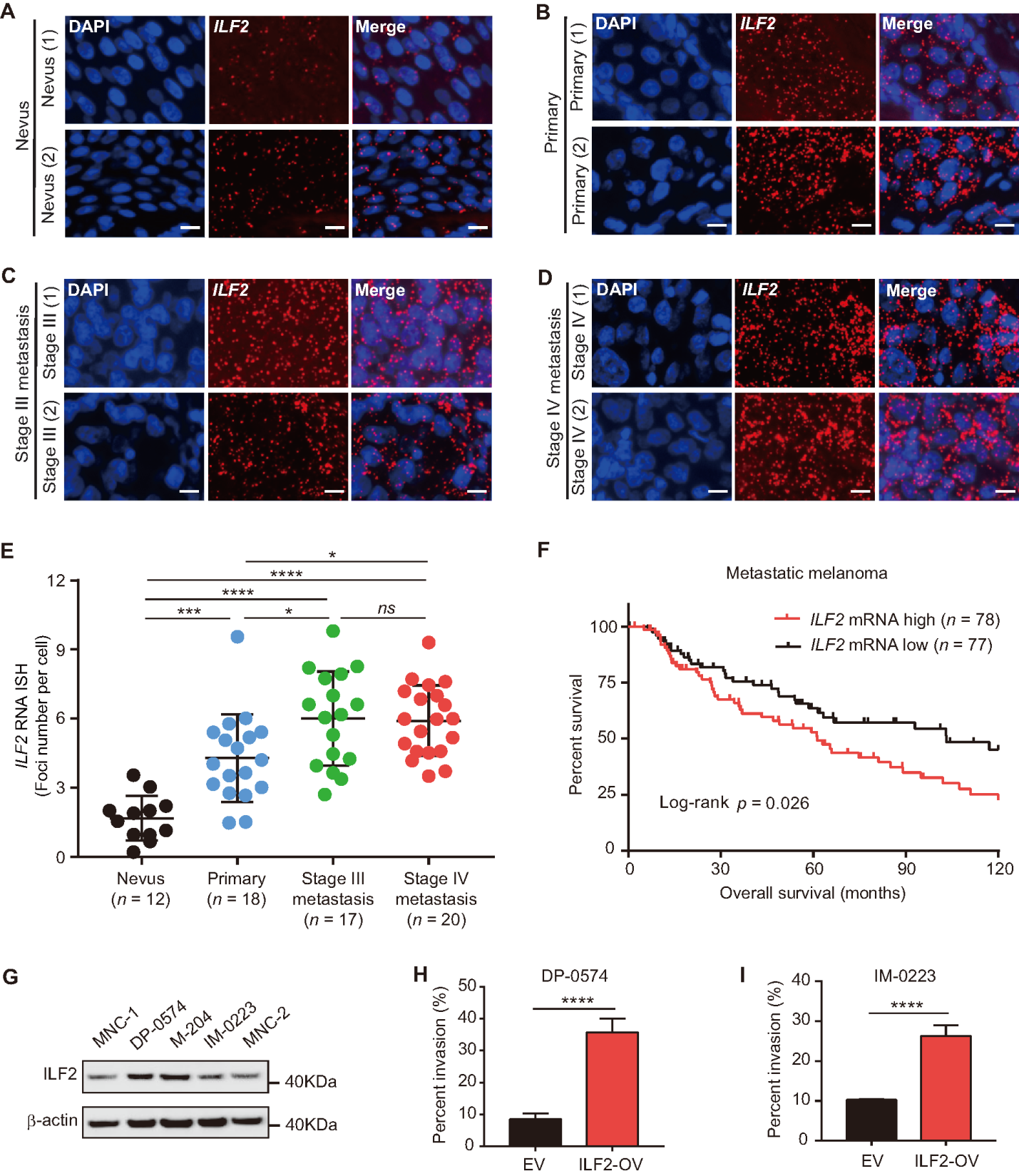


**FIGURE S1.** Assessment of *ILF2* mRNA and protein expression in melanoma. (**A**-**E**) Representative tissue images (**A**-**D**) and *ILF2* mRNA quantification (**E**) using RNA ISH in FFPE tissue samples from nevus, primary melanoma, and stage III and IV melanoma metastasis. Scale bars = 10 µm. (**F**) Kaplan-Meier curve for OS in metastatic melanoma patients that were divided according to *ILF2* mRNA expression into lower (*n* = 77) and upper quartile (*n* = 78) using the TCGA SKCM dataset. (**G**) Western blot for ILF2 in DP-0574, M-204, IM-0223, and two melanocytes (MNC; MNC-1 and MNC-2) cell lines. β-actin was used as the loading sample control. (**H** and **I**) The percent of invasion for DP-0574 (**H**) and IM-0223 (**I**) melanoma cell lines with ILF2-OV compared to respective EV control cell lines. Data represent the mean ± SD. *ns*: not significant, **p* < 0.05, ****p* < 0.001, and *****p* < 0.0001.

**
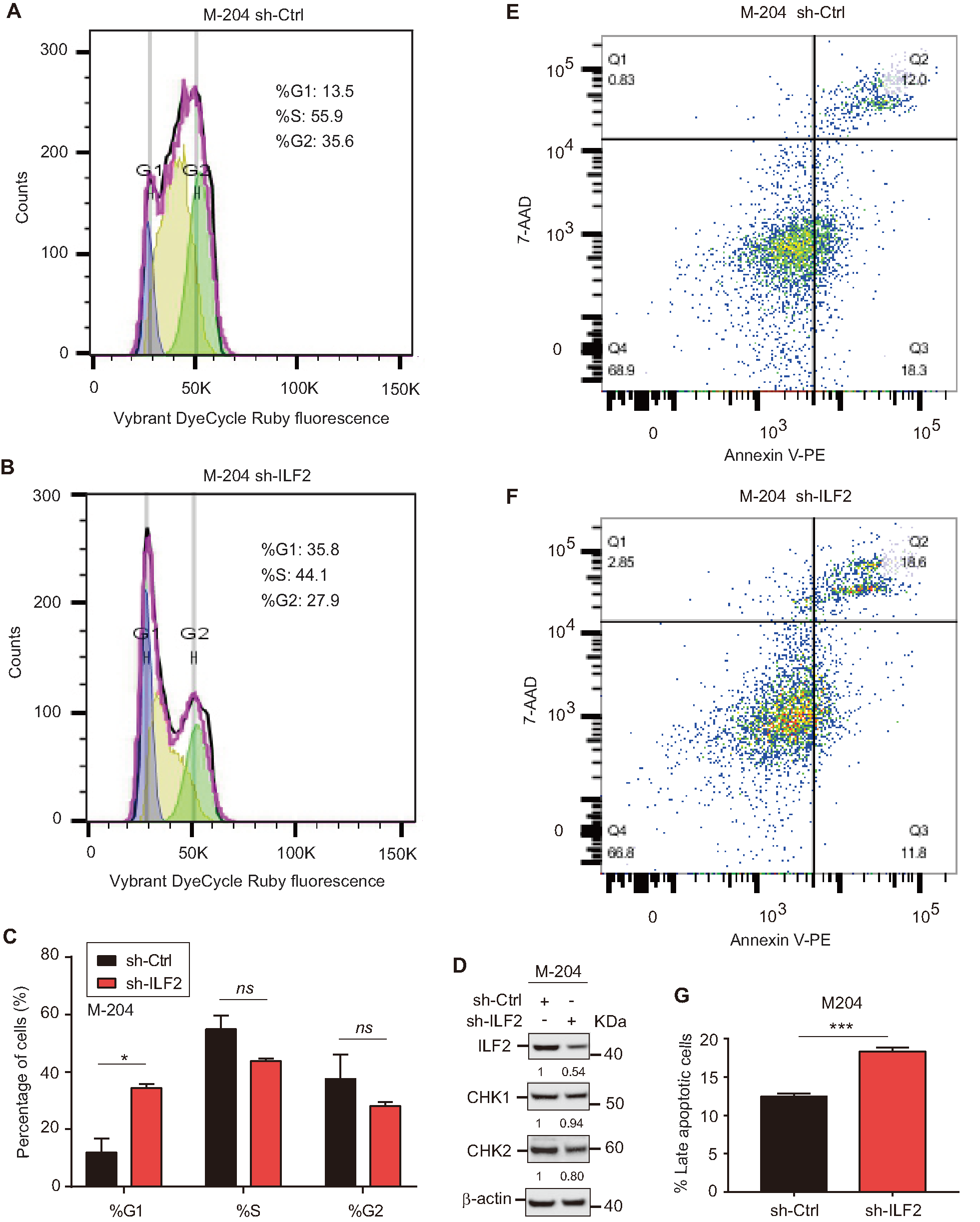
**

**FIGURE S2.** ILF2 knockdown blocked cell cycle and induced apoptosis in melanoma cells. (**A**-**C**) Representative images (**A** and **B**) and quantification (**C**) of cell cycle analysis in M-204 sh-Ctrl and sh-ILF2 cells. (**D**) Western blot and quantification for ILF2, CHK1, and CHK2 in M-204 sh-Ctrl and sh-ILF2 cell lines. β-actin was used as the loading sample control. (**E**-**G**) Representative images (**E** and **F**) and quantification (**G**) of apoptosis analysis in M-204 sh-Ctrl and sh-ILF2 cells. Data represent the mean ± SD. *ns*: not significant, **p* < 0.05, and ****p* < 0.001.

**
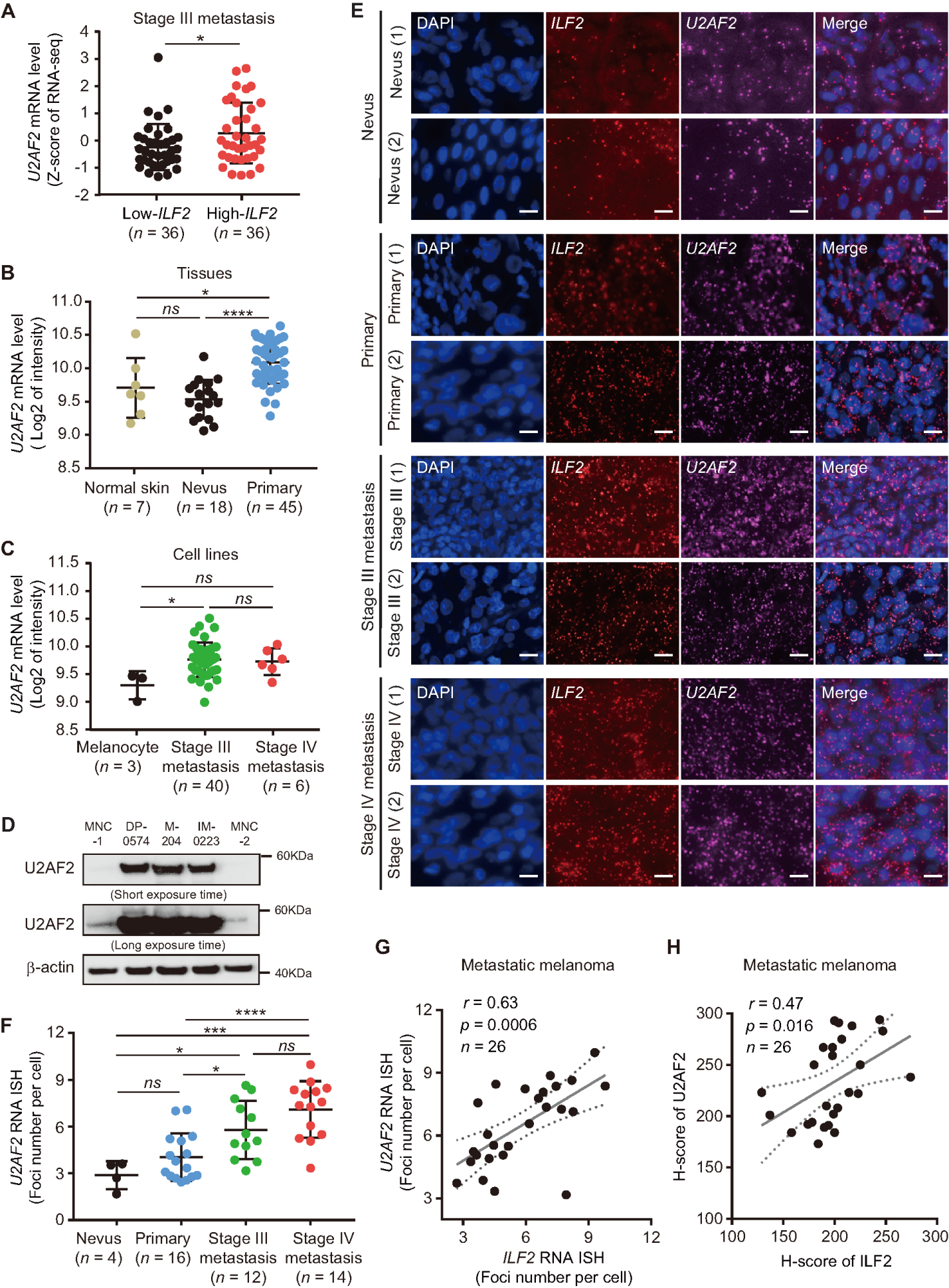
**

**FIGURE S3.** *U2AF2* is highly expressed in metastatic melanoma and positively correlated with *ILF2* expression. (**A**) Comparison of *U2AF2* mRNA expression in patients with low *ILF2* and high *ILF2* mRNA expression. Groups were defined by ILF2 quartile expression obtained from TCGA SKCM dataset. (**B**) Comparison of *U2AF2* mRNA expression in normal skin, nevus, and primary melanoma samples using GSE3189 microarray dataset. (**C**) Comparison of *U2AF2* mRNA expression in melanocytes (MNC), stage III metastasis, and stage IV metastasis melanoma lines from the SJCI microarray dataset. (**D**) Western blot for U2AF2 in DP-0574, M-204, IM-0223, and two melanocytes (MNC, MNC-1 and MNC-2) cell lines with short- and long-exposure times. β-actin was used as the loading control. (**E**) Representative images of RNA ISH using *ILF2* (red) and *U2AF2* (magenta) RNA probe for nevus, primary, stage III metastasis, and stage IV metastasis melanoma FFPE samples. Scale bars = 10 µm. (**F**) RNA ISH quantitation of *U2AF2* in nevus, primary, stage III metastasis, and stage IV metastasis melanoma FFPE samples. (**G**) Correlation between *ILF2* and *U2AF2* mRNA levels determined by RNA ISH assay. (**H**) Correlation between ILF2 and U2AF2 protein levels using the H-score values determined by IHC assay in metastatic melanoma FFPE samples. Data represent the mean ± SD. *ns*: not significant, **p* < 0.05, ****p* < 0.001, and *****p* < 0.0001. The correlation of ILF2 and U2AF2 mRNA or protein expression was determined by Pearson’s (**G** and **H**) correlation test.

**
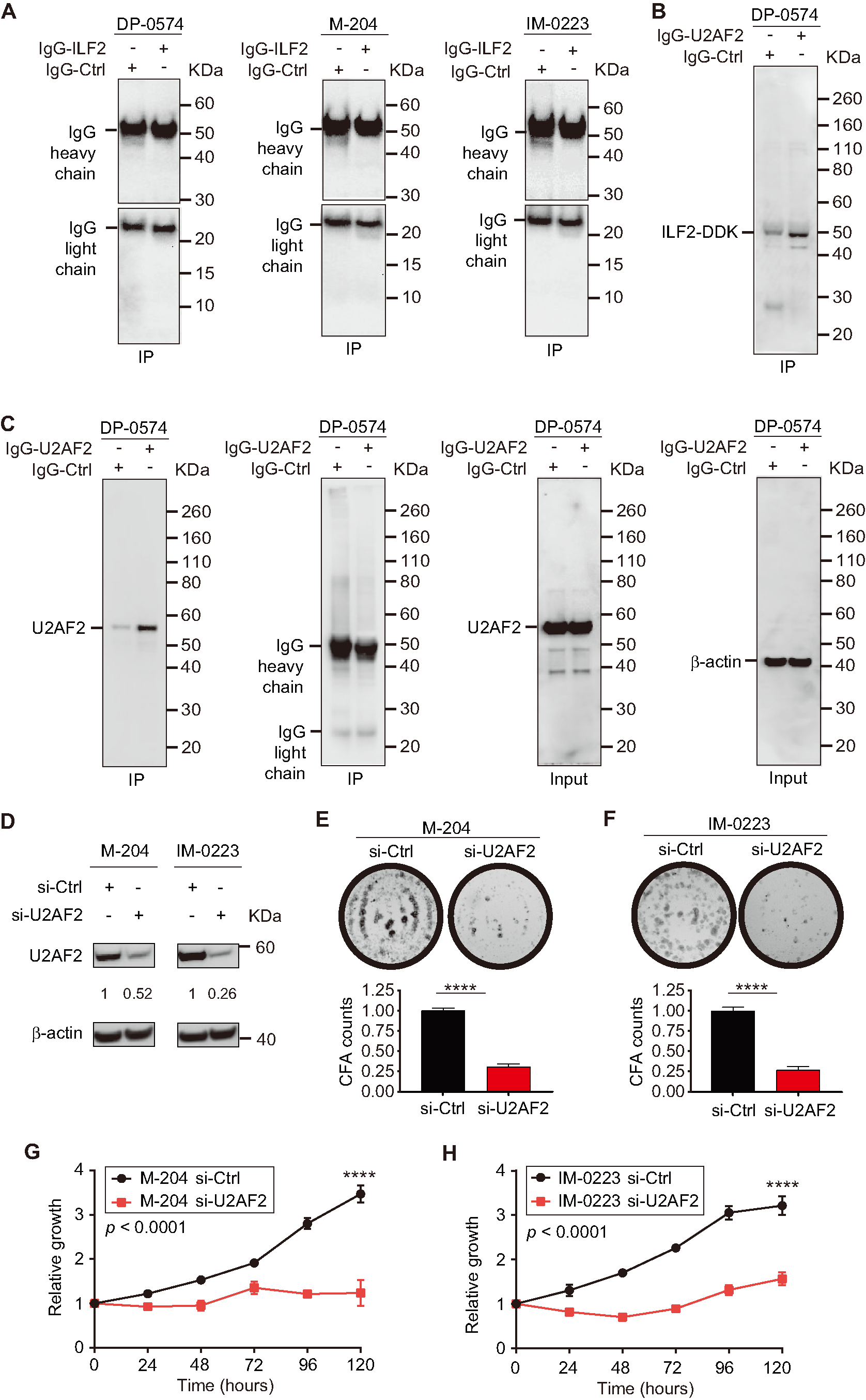
**

**FIGURE S4.** U2AF2 knockdown reduces colony formation and cell proliferation. (**A**) Western blot for IgG heavy and light chains controls for Figure 4B Co-IP assays. (**B** and **C**) Western blot for ILF2-DDK, U2AF2, and IgG chains in reciprocal Co-IP assays using IgG U2AF2 antibody or IgG control. U2AF2 and β-actin proteins were assessed in corresponding input samples. (**D**) Western blot and the quantification of U2AF2 in M-204 and IM-0223 cell lines transfected with si-Ctrl or si-U2AF2. β-actin was the loading control. (**E** and **F**) Representative colony formation images and the quantification of M-204 (**E**) and IM-0223 (**F**) cell lines transfected with si-Ctrl or si-U2AF2. (**G** and **H**) Proliferation assays of M-204 (**G**) or IM-0223 (**H**) cells transfected with si-Ctrl or si-U2AF2. Data represent the mean ± SD. *****p* < 0.0001.

**
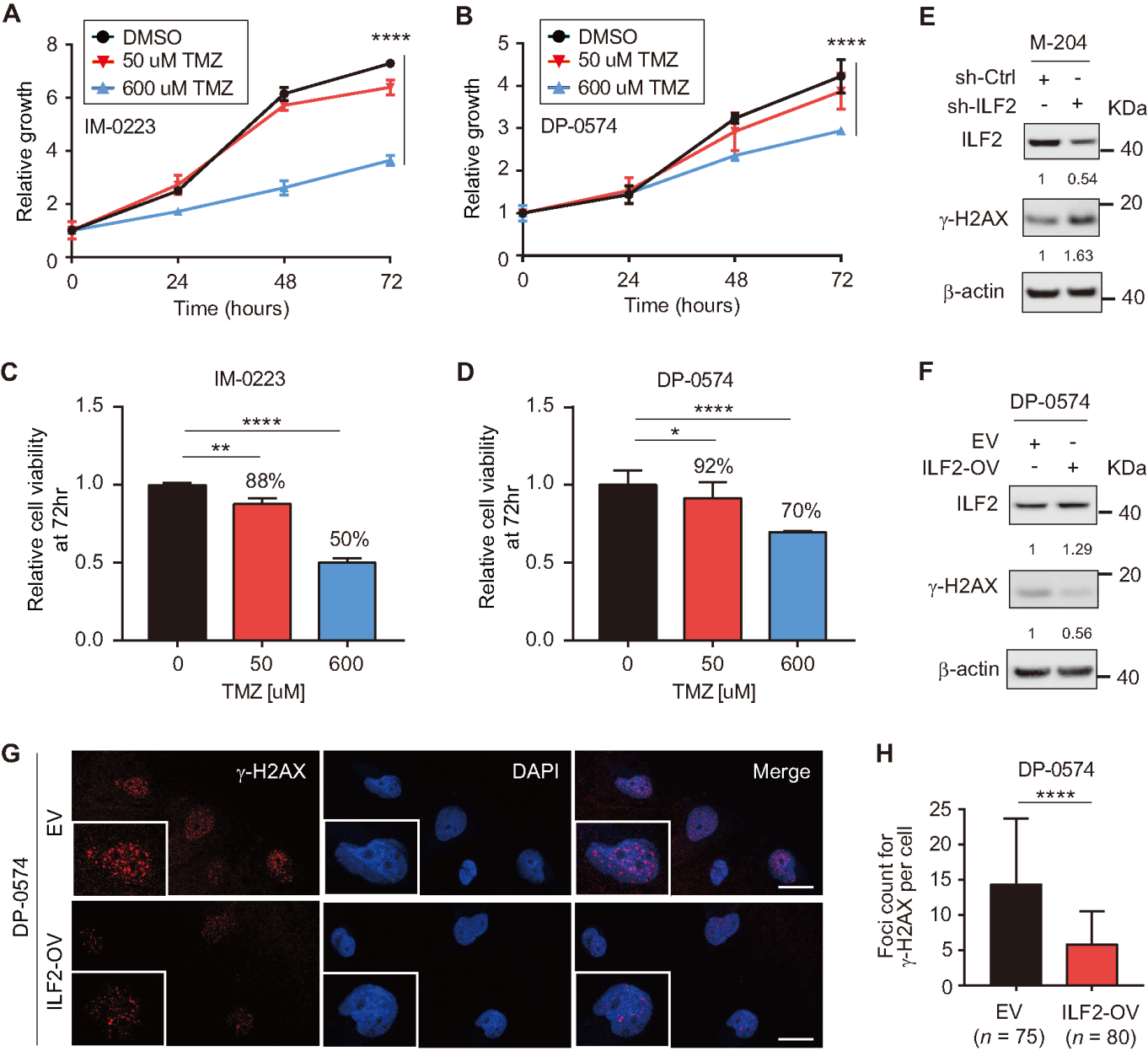
**

**FIGURE S5.** DNA damage response to Temozolomide in melanoma cell lines. (**A** and **B**) Temozolomide (TMZ) sensitivity assays for IM-0223 (**A**) and DP-0574 (**B**) cell lines treated with DMSO as control, 50 µM, or 600 µM TMZ for 24, 48, and 72 hours. (**C** and **D**) Relative growth at 72 hours in IM-0223 (**C**) and DP-0574 (**D**) cell lines treated with DMSO, 50 µM, or 600 µM TMZ. (**E** and **F**) Western blot and the quantification of ILF2 and γ-H2AX in melanoma cells with ILF2 knockdown (**E**) or overexpression (**F**). β-actin was used as the loading control. (**G** and **H**) Representative confocal images (**G**) and foci quantification (**H**) for γ-H2AX immunofluorescence staining in DP-0574 EV and ILF2-OV cells. Scale bars = 10 µm. Data represent the mean ± SD. **p* < 0.05, ***p* < 0.01, and *****p* < 0.0001.


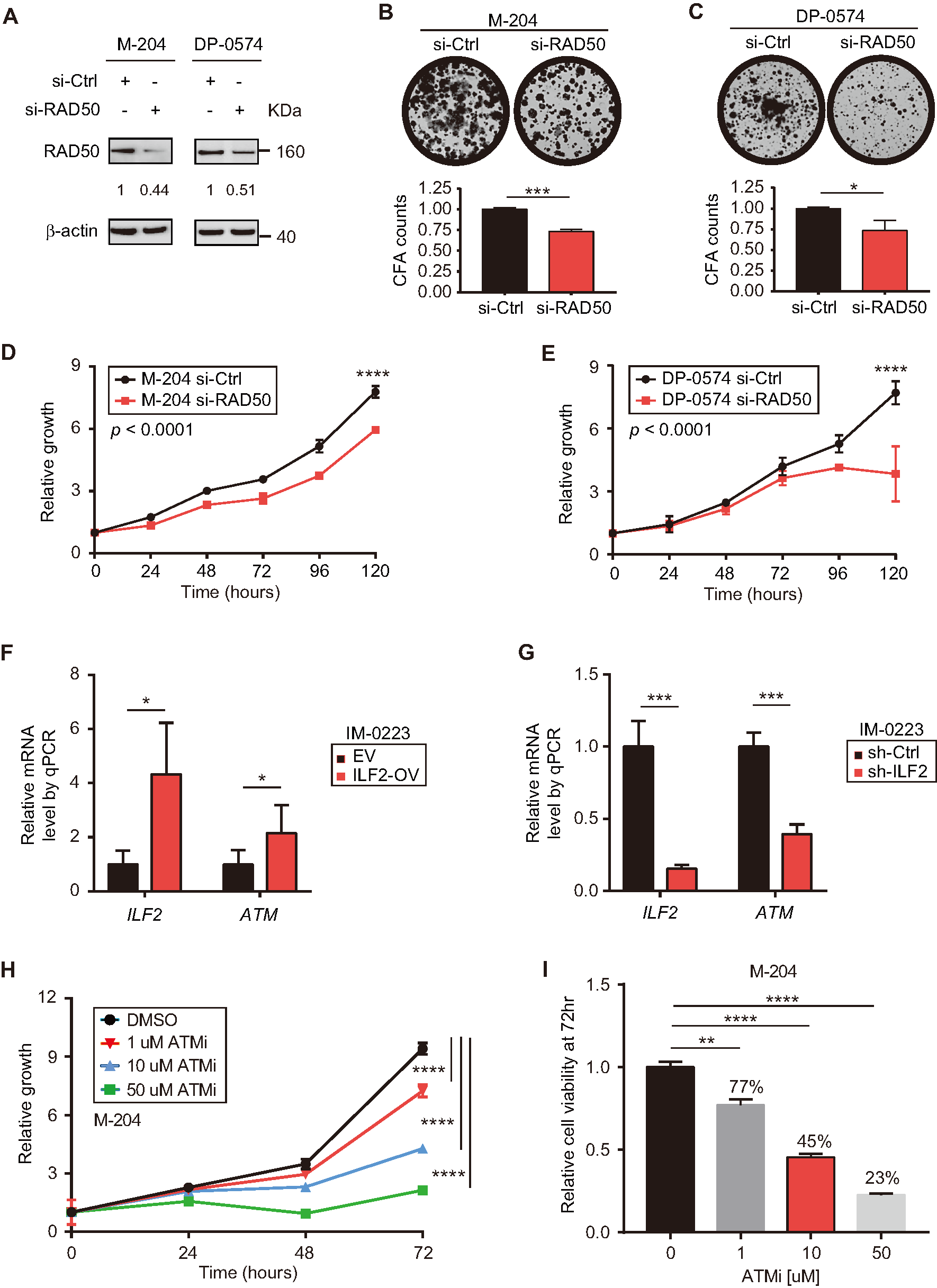


**FIGURE S6.** ILF2 regulates ATM pathway. (**A**) Western blot and the quantification of RAD50 in M-204 and DP-0574 cell lines transfected with si-Ctrl or si-RAD50. β-actin was the loading sample control. (**B** and **C**) Representative colony formation images and the quantification of M-204 (**B**) and DP-0574 (**C**) cell lines transfected with si-Ctrl or si-RAD50. (**D** and **E**) Proliferation assays using M-204 (**D**) or DP-0574 (**E**) cell lines transfected with si-Ctrl or si-RAD50. (**F** and **G**) RT-qPCR assay analysis for *ILF2* and *ATM* mRNA expression in ILF2 overexpressed (**F**) or knockdown (**G**) melanoma cell lines. (**H**) Sensitivity assays in M-204 cell lines using increasing concentrations of the ATM inhibitor (ATMi) 1 µM, 10 µM, or 50 µM or DMSO as control, for 24, 48, and 72 hours. (**I**) Relative growth at 72 hours in M-204 cells treated with DMSO, 1 µM, 10 µM, or 50 µM ATMi. Data represent the mean ± SD. **p* < 0.05, ***p* < 0.01, ****p* < 0.001, and *****p* < 0.0001.


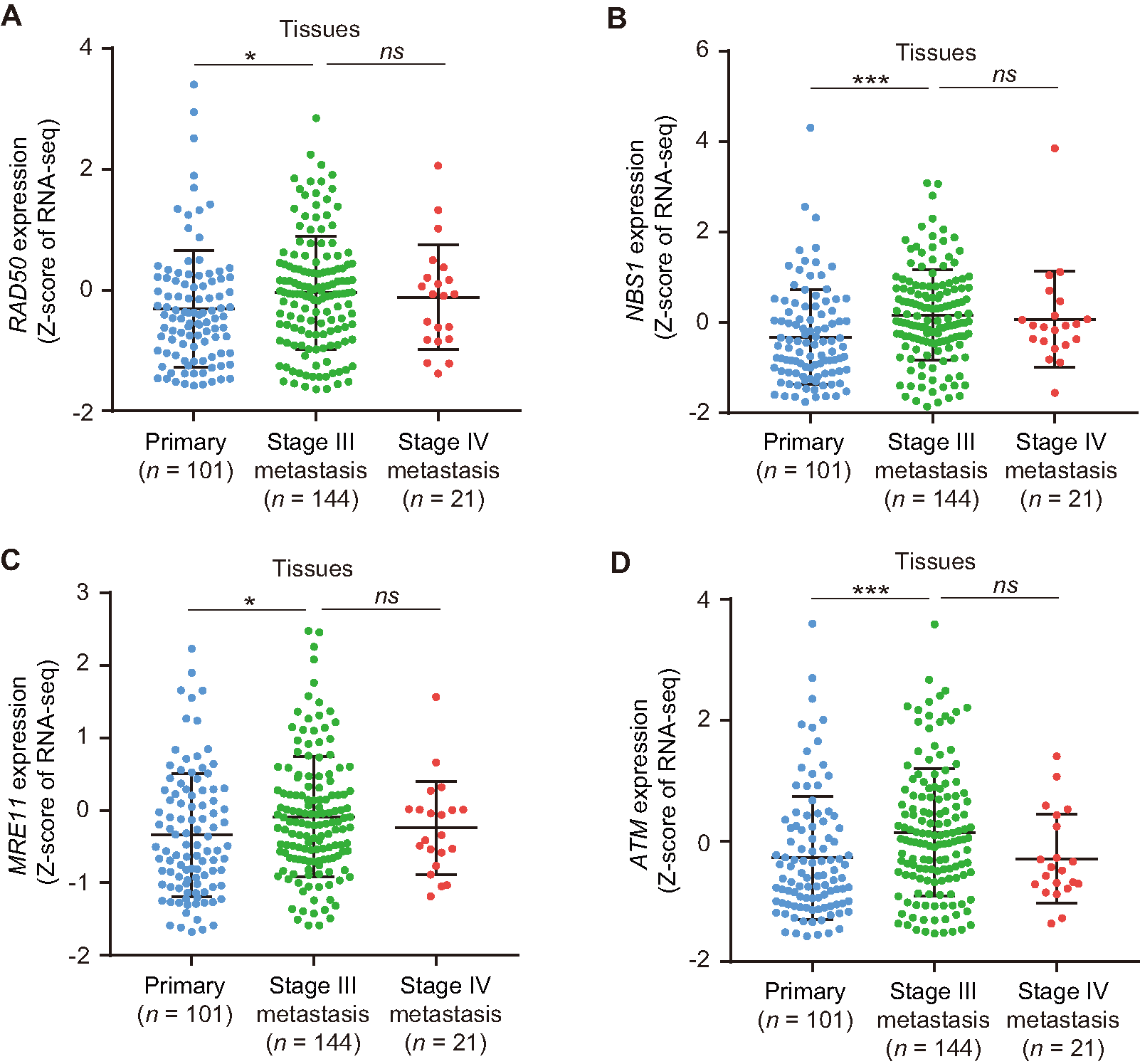


**FIGURE S7.** *RAD50*, *NBS1*, *MRE11,* and *ATM* mRNA expression are upregulated in metastatic melanoma. (**A**-**D**) Comparison of *RAD50*, *NBS1*, *MRE11*, or *ATM* mRNA expression in primary, stage III metastasis, and stage IV metastatic melanoma tissues using the TCGA SKCM RNA-seq dataset. Data represent the mean ± SD. *ns*: not significant, **p* < 0.05, and ****p* < 0.001.


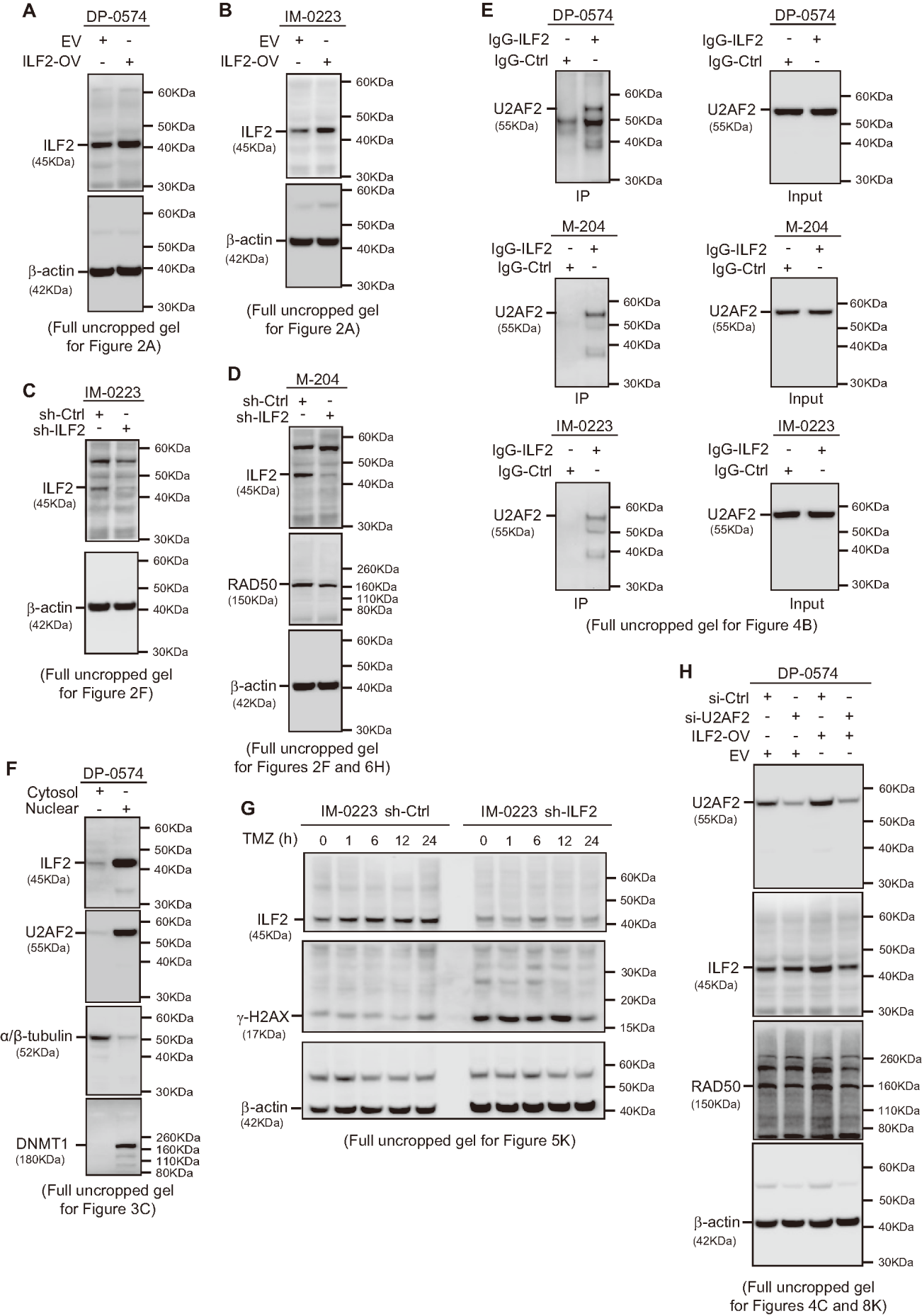


**FIGURE S8.** Uncropped western blot images for Figures **2A**, **2F**, **6H**, **4B**, **3C**, **5K**, **4C**, and **8K**.


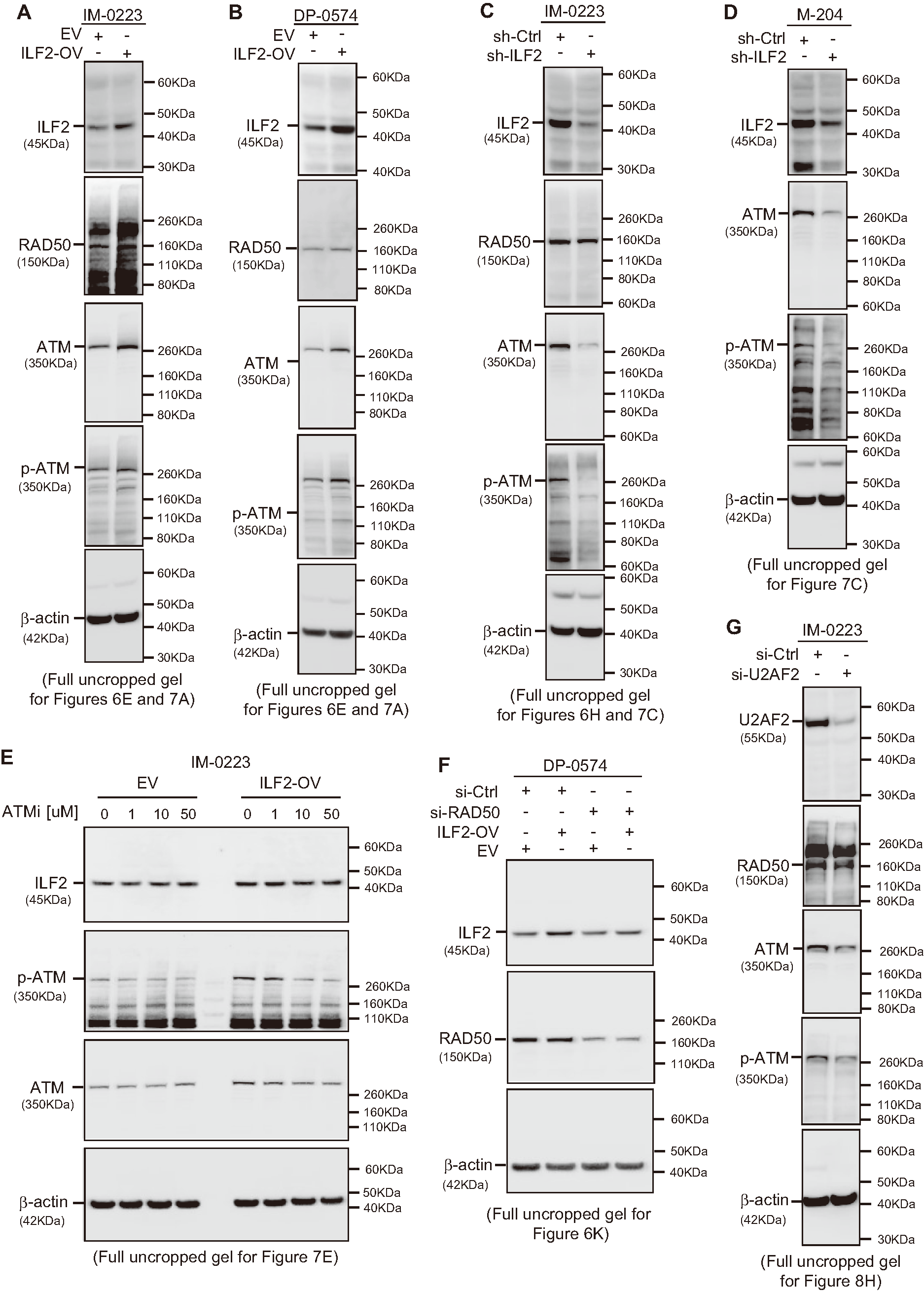


**FIGURE S9.** Uncropped western blot images for Figures **6E**, **7A**, **6H**, **7C**, **7E**, **6K**, and **8H**.


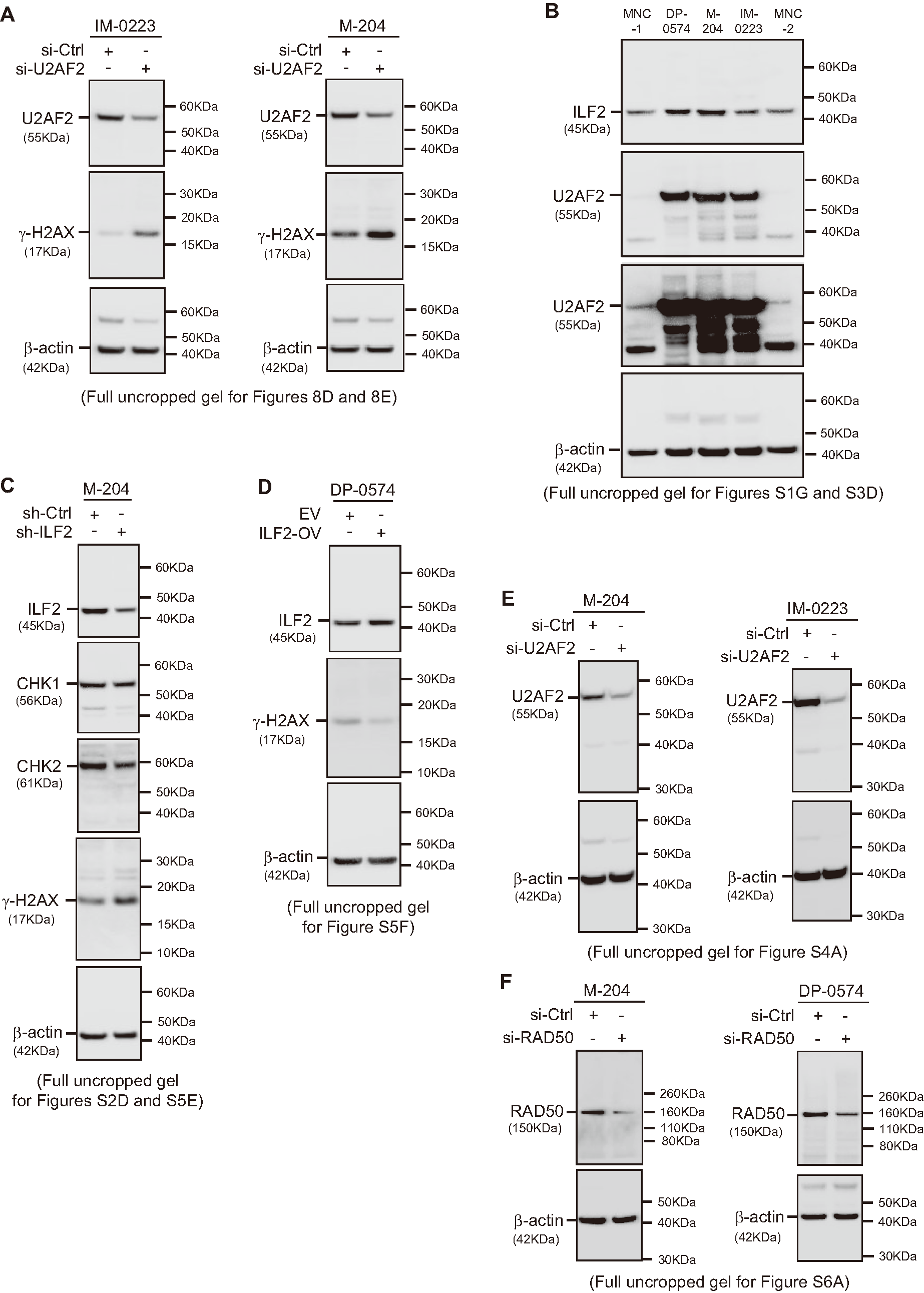


**FIGURE S10.** Uncropped western blot images for Figures **8D**, **8E**, **S1G**, **S3D**, **S2D**, **S5E**, **S5F**, **S4A**, and **S6A**.

**TABLE S1. Clinical pathology information for melanoma patients included in the study**

|  | **Melanoma patients**  ^†^**IHC (*n* = 80)** | **Melanoma patients**  **RNA** ^‡^**ISH (*n* = 55)** |
| --- | --- | --- |
| **Variables** | ***n* (%)** | ***n* (%)** |
| Age at diagnosis, mean (^§^SD) | 52.4 (17.8) | 56.5 (20.2) |
| <60 | 47 (58.7) | 26 (47.3) |
| ≥60 | 33 (41.3) | 29 (52.7) |
| Gender |  |  |
| Male | 53 (66.3) | 36 (65.5) |
| Female | 27 (33.7) | 19 (34.5) |
| ^¶^AJCC pathological stages |  |  |
| I/II | 56 (70) | 35 (63.6) |
| III | 20 (25) | 18 (32.7) |
| IV | 4 (5) | 2 (3.7) |
| Tumor location |  |  |
| Primary | 22 (27.5) | 18 (32.7) |
| Lymph node metastasis | 12 (15) | 17 (30.9) |
| Distant metastasis | 46 (57.5) | 20 (36.4) |

^†^IHC = immunohistochemistry.

^‡^ISH = in-situ hybridization.

^§^SD = standard deviation.

^¶^AJCC stage = American Joint Committee on Cancer at melanoma diagnosis.

**TABLE S2. Key resources used in this study**

| **REAGENT or RESOURCE** | | **SOURCE** | | **IDENTIFIER** |
| --- | --- | --- | --- | --- |
| Antibodies | | | | |
| Mouse monoclonal anti-ILF2 | | Santa Cruz | | Cat# sc-365068 |
| Mouse monoclonal anti-U2AF2 | | Santa Cruz | | Cat# sc-53942 |
| Mouse monoclonal anti-β-actin | | Sigma-Aldrich | | Cat# A5441 |
| Rabbit polyclonal anti-γ-H2AX | | Novus Biologicals | | Cat# NB100-2280 |
| Rabbit polyclonal anti-RAD50 | | Cell Signaling Technology | | Cat# 3427 |
| Rabbit monoclonal anti-NBS1 | | Cell Signaling Technology | | Cat# 14956 |
| Rabbit monoclonal anti-MRE11 | | Cell Signaling Technology | | Cat# 4895 |
| Rabbit monoclonal anti-ATM | | Cell Signaling Technology | | Cat# 2873 |
| Rabbit monoclonal anti-phospho-ATM | | Cell Signaling Technology | | Cat# 5883 |
| Rabbit polyclonal anti-DNMT1 | | Novus Biologicals | | Cat# NB100-264 |
| Rabbit polyclonal anti-α/β-Tubulin | | Cell Signaling Technology | | Cat# 2148 |
| Rabbit IgG normal | | Cell Signaling Technology | | Cat# 2729 |
| Rabbit polyclonal anti-ILF2 | | Abcam | | Cat# ab113205 |
| Rabbit polyclonal anti-U2AF2 | | Abcam | | Cat# ab37530 |
| Recombinant ILF2-DDK protein | | Origene | | Cat# TP301751 |
| Mouse monoclonal anti-DDK | | Origene | | Cat# OTI4C5 |
| Mouse monoclonal anti-CHK1 | | Cell Signaling Technology | | Cat# 2360 |
| Rabbit polyclonal anti-CHK2 | | Cell Signaling Technology | | Cat# 2662 |
| Bacterial and Virus Strains | | | | |
| ILF2 ORF cDNA lentiviral particles | | GeneCopoeia | | Cat# LPP-T0515-lv205-100 |
| Negative control lentiviral particles | | GeneCopoeia | | Cat# LPP-NEG-lv105-025-C |
| ILF2 shRNA lentiviral particles | | Dharmacon | | Cat# VGH5518-200158084 |
| Non-silencing shRNA control lentiviral particles | | Dharmacon | | Cat# RHS4348 |
| Biological Samples | |  | |  |
| Melanoma patients FFPE tissues | | SJCI pathology | | Pathology Department. Saint John’s Health Center at Providence Health System |
| Chemicals, Peptides, and Recombinant Proteins | | | | |
| Temozolomide | | Selleck Chemicals | | Cat# S1237 |
| Puromycin dihydrochloride | | Life Technologies | | Cat# A1113803 |
| KU-55933 (ATM kinase inhibitor) | | Selleck Chemicals | | Cat# 1092 |
| DAPI | | Life Technologies | | Cat# 62248 |
| Texas-Red-X Phalloidin | | Life Technologies | | Cat# T7471 |
| Hs-ILF2-C2 probe | | Advanced Cell Diagnostics | | Cat# 557851-C2 |
| Hs-U2AF2-C3 probe | | Advanced Cell Diagnostics | | Cat# 575271-C3 |
| Critical Commercial Assays | | | | |
| CellTiter-Glo^®^ Luminescent Cell Viability Assay | | Promega | | Cat# G7572 |
| JetPRIME^TM^ Transfection Reagent | | VWR | | Cat# 89129-924 |
| Pierce^TM^ BCA Protein Assay Kit | | Life Technologies | | Cat# 23227 |
| Quick-gDNA^TM^ MiniPrep Kit | | Zymo Research | | Cat# D3025 |
| RNAscope^®^ Multiplex Fluorescent Reagent Kit | | Advanced Cell Diagnostics | | Cat# 323100 |
| Nuclear Extract Kit | | Active Motif | | Cat# 40010 |
| Homologous Recombination Assay Kit | | Norgen Biotek | | Cat# 35600 |
| BME Cell Invasion Kit | | Cultrex | | Cat# 3455-096-K |
| Deposited Data | | | | |
| RPPA data (DP-0574 and IM-0223 cell lines) | | This paper | | GSE159692 |
| Primary malignant melanoma microarray data | | Public data | | GSE3189 |
| Melanoma metastasis microarray data | | Public data | | GSE8401 |
| SJCI melanoma cell lines microarray data | | Public data | | GSE44662 |
| Experimental Models: Cell Lines | | | | |
| Human DP-0574 cell line | | This paper | | SJCI |
| Human IM-0223 cell line | | This paper | | SJCI |
| Human M-204 cell line | | This paper | | SJCI |
| Oligonucleotides | | | | |
| ON-TARGETplus Human U2AF2 siRNA | | Dharmacon | | Cat# L-012380-02-0005 |
| ON-TARGETplus Human RAD50 siRNA | | Dharmacon | | Cat# L-005232-00-0005 |
| ON-TARGETplus Non-targeting pool siRNA | | Dharmacon | | Cat# D-001810-10-05 |
| Softwares and Algorithms | | | | |
| ImageJ | | NIH | | <https://imagej.nih.gov/ij/> |
| GraphPad Prism 7 | | GraphPad Software | | [www.graphpad.com](http://www.graphpad.com) |
| Adobe Illustrator | | Adobe | | [www.adobe.com](http://www.adobe.com) |
| Mantra Snap 1.03 | | Perkin Elmer | | [www.perkinelmer.com](http://www.perkinelmer.com) |
| Primer Sequences (5’-3’) for qPCR | | | | |
| ILF2 | F: TTTTAAGGCGCCATGAGGGG | | R: GGGAAAGGCCATTTCACACAAAT | |
| U2AF2 | F: AAGAATGCCACGCTGAGCAC | | R: AGCACCATGTTCATGAGGCAC | |
| RAD50 | F: CTTATACAGGACCAGCAGGAAC | | R: GCTGACGACGTTGCAAATTA | |
| ATM | F: GCACAGAAGTGCCTCCAATTC | | R: ACATTCTGGCACGCTTTGG | |
| SDHA | F: TCAGCATGCAGAAGTCAAT | | R: GAACG TCTTCAGGTGCTTT | |
